# Curcumin affects gene expression and reactive oxygen species via a PKA dependent mechanism in *Dictyostelium discoideum*

**DOI:** 10.1371/journal.pone.0187562

**Published:** 2017-11-14

**Authors:** William S. Swatson, Mariko Katoh-Kurasawa, Gad Shaulsky, Stephen Alexander

**Affiliations:** 1 Division of Biological Sciences, University of Missouri, Columbia, MO, United States of America; 2 Department of Molecular and Human Genetics, Baylor College of Medicine, Houston, TX, United States of America; Université de Genève, SWITZERLAND

## Abstract

Botanicals are widely used as dietary supplements and for the prevention and treatment of disease. Despite a long history of use, there is generally little evidence supporting the efficacy and safety of these preparations. Curcumin has been used to treat a myriad of human diseases and is widely advertised and marketed for its ability to improve health, but there is no clear understanding how curcumin interacts with cells and affects cell physiology. *D*. *discoideum* is a simple eukaryotic lead system that allows both tractable genetic and biochemical studies. The studies reported here show novel effects of curcumin on cell proliferation and physiology, and a pleiotropic effect on gene transcription. Transcriptome analysis showed that the effect is two-phased with an early transient effect on the transcription of approximately 5% of the genome, and demonstrates that cells respond to curcumin through a variety of previously unknown molecular pathways. This is followed by later unique transcriptional changes and a protein kinase A dependent decrease in catalase A and three superoxide dismutase enzymes. Although this results in an increase in reactive oxygen species (ROS; superoxide and H_2_O_2_), the effects of curcumin on transcription do not appear to be the direct result of oxidation. This study opens the door to future explorations of the effect of curcumin on cell physiology.

## Introduction

The use of botanicals as dietary supplements is becoming increasingly popular. The World Health Organization (WHO) estimates that 80% of the world’s population uses botanicals as part of their primary health care. In the United States, 20% of Americans use botanicals, with billions of dollars spent each year on these products [1]. The global botanical market was valued at $54.6 billion dollars in 2013 with a forecasted market value estimated to reach $90.2 billion by 2020 [2].

Curcumin is the active ingredient in turmeric [3] and has been widely used in traditional medicine, especially in India, China and Thailand. It has been used to treat many diseases including anorexia, cough, diabetic wounds, hepatic disorder, rheumatism and sinusitis [4]. Curcumin has been linked to a wide spectrum of pharmacological effects including anti-carcinogenic, anti-inflammatory, Alzheimer’s prevention and antioxidant activity [5]. It has also been implicated in controlling the growth, development and spread of cancer by interfering with the cell cycle, apoptosis, proliferation, angiogenesis and metastasis [6, 7].

Chronic low-level inflammation has been associated with many diseases including heart disease [8], metabolic syndrome [9] and cancer [10, 11]. Some studies have implicated curcumin in blocking NF-kB, a transcription factor that plays a key role in turning on genes related to inflammation [12]. Indeed, studies have indicated that curcumin has higher efficacy and fewer side effects compared to other anti-inflammatory drugs [13, 14]. Alzheimer’s disease, the most common neurodegenerative disease in the world, is characterized by a buildup of protein tangles in the brain known as amyloid plaques, and studies have shown that curcumin has a potential role in the prevention of the disease [15] and even the ability to clear amyloid plaques by enhancing the surface binding of amyloid-beta to macrophages and intracellular phagocytosis leading to amyloid-beta degradation [16].

An antioxidant role is often ascribed to curcumin. Oxidative damage, the reaction of free radicals with cellular lipids, proteins and DNA, is associated with the cause of many diseases and aging. Reactive oxygen species (ROS), such as superoxide anions, peroxides and hydroxyl radicals, are by-products of aerobic metabolism, and their accumulation can lead to oxidative stress and significant damage to cells [17, 18]. Because of the potentially damaging effects of ROS, cells use enzymes such as superoxide dismutase, peroxidase and catalase as antioxidant defense mechanisms. [19]. Other non-enzymatic molecules capable of inhibiting ROS include vitamin E and uric acid [20–22]. Curcumin has been reported to have antioxidant effects [23] in rat peritoneal macrophages [22] and in red blood cells [24].

Despite the large and growing interest in the use of botanicals in disease treatment and prevention, there is little evidence regarding their efficacy, safety and long-term effects. Importantly, the fundamental mechanisms associated with the cellular response to botanicals are generally not understood, and there are often unknown off-target effects. Curcumin has been associated with a large number of effects but reports have been baffling and sometimes contradictory. Some evidence suggests that curcumin is an antioxidant, whereas other studies suggest that curcumin has pro-oxidant effects, increasing ROS and thereby inducing oxidative stress within cells [25, 26]. Possessing both antioxidant and pro-oxidant effects confounds the rational therapeutic use of curcumin. In addition a recent review indicates that clinical trials of curcumin against various diseases have been unsuccessful due in part to issues of bio-availability and the stability of curcumin in aqueous solution [27]. However, curcumin or its breakdown products/derivatives do have effects on cell physiology, although there is a lack of understanding of the underlying mechanisms of action at the cellular level. Thus, a better understanding of the molecular mechanisms underlying the response to curcumin is required to validate its pharmacological use in human health and would benefit from a genetically tractable model system that would be useful to interrogate the molecular effects of curcumin on cell physiology.

The social amoeba *Dictyostelium discoideum* is an excellent model for the molecular genetic study of the mechanisms of action of natural products [28, 29] and drugs and their effects on cells [30, 31]. *D*. *discoideum* is a haploid eukaryote which proliferates rapidly as single cells. Its cell structure and physiology are similar to mammalian cells and have been intensely studied [32]. Moreover, it is a genetic system that has been readily validated and translated to human cells [33, 34]. Therefore, *D*. *discoideum* offers a useful lead genetic system with which to study the underlying mechanisms of action of curcumin.

The current study is focused on the effect of curcumin on cell viability and proliferation. The study shows that curcumin has profound pleiotropic effects on the physiology of mitotically dividing *D*. *discoideum* cells, including an accumulation of ROS, which is affected by down-regulation of antioxidant gene expression. This transcriptionally regulated physiological response is mediated, in part, by the cyclic AMP-dependent protein kinase A (PKA). RNA sequencing (RNA-seq) analysis reveals a broad, two-phase, transcriptional response elicited by curcumin and suggests that cells respond to curcumin through a variety of previously unknown molecular pathways.

Taken together, these studies highlight the relative lack of understanding about the effect of curcumin (and by extension, other botanicals) on genetic regulation and cell physiology. Importantly, they provide new avenues of investigation that can be further interrogated using the genetic tools of *D*. *discoideum* and then translated in human cells as has been previously done in studies on other medically important drugs [33, 34].

## Materials and methods

### Reagents

Chemicals were from Sigma-Aldrich (St. Louis, MO) and Fisher Scientific (Pittsburgh, PA). Curcumin stock solutions were prepared in DMSO and the concentration was determined by measuring the absorbance at 423 nm. The stock solutions were then adjusted to 50 mg/ml using the empirically determined extinction coefficient ε = 54954 cm^-1^ M^-1^. Sub-stock solutions were made by dilution in DMSO so that curcumin was always delivered in the same volume yielding a final DMSO concentration of 0.2% which has no effect on *D*. *discoideum* cell proliferation. Stock solutions of menadione (50 mg/ml in H_2_O) and N-acetylcysteine (NAC; 100 mg/ml in H_2_O) were prepared. All stock solutions were stored frozen at -20°C and warmed to room temperature before delivery. Control cultures received the same concentration of vehicle.

### Strains and growth conditions

The *D*. *discoideum* strains used in this study are listed in S1 Table along with their genotypes, phenotypes and sources. The cells were grown axenically at 22°C in shaken HL5 medium [35]. New cultures were started monthly from desiccated spores. Cell number was monitored by multiple counts in a hemocytometer. Stock cells were always kept in log-phase growth and never allowed to grow beyond 5x10^6^ cells/ml. Logarithmically growing cells at a density of 2-4x10^6^ cells/ml in HL5 medium were used for experiments and had a generation time of 10–12 hours.

### Measurement of cell proliferation and viability

Cell proliferation measurements employed a luminescent cell viability method used to determine the number of viable cells based on the amount of ATP in metabolically active cells [36]. For each curcumin concentration to be tested, the parent culture (2-4x10^6^ cells/ml) was diluted to 1x10^5^ cells/ml in 5 ml aliquots, after which compounds or vehicle was added. The CellTiter-Glo® luminescent cell viability assay (Promega, Madison, WI) was performed in opaque 96 well plates (Thermo Scientific, Waltham, MA). At the indicated times, 100 μl of treated or control cells were sampled and mixed with an equal volume of CellTiter-Glo® for 10 minutes at 22°C. Luminescence was measured using a Veritas microplate luminometer (Turner BioSystems, Sunnyvale, CA). All experiments were done in triplicate, and were always in the linear range of the technique [36]. To test clonal viability, treated or control cells were plated on SM agar plates in association with *Klebsiella aerogenes*. Multiple plating at different dilutions was performed to ensure that the counts were in the linear range of this technique.

### Catalase A activity assay

Catalase enzyme activity was determined as described [37]. Dividing cells in axenic medium (10 ml in 125 ml flasks) were treated with compounds at the indicated concentration, harvested and washed by centrifugation in salts solution (SS; 10 mM NaCl, 10 mM KCl, 2.7 mM CaCl_2_). The cells were pelleted and then lysed in 100 μl lysis buffer (10 mM potassium phosphate, pH 7.0, 0.1% Triton X-100, 1x protease inhibitor cocktail (Sigma-Aldrich, St. Louis, MO)) for 5 minutes on ice. Samples of 5 μl and 10 μl of the cell lysate were assayed for catalase activity by mixing with 10 mM H_2_O_2_ in 50 mM potassium phosphate buffer, pH 7.0 in a final volume of 1 ml. The reduction of absorbance at 240 nm was monitored over a two-minute period at 10-second intervals with a spectrophotometer [37]. There is no non-catalytic degradation of H_2_O_2_ [35]. One absorbance unit equals 1 μmole of H_2_O_2_ using an extinction coefficient of 43.6 M^-1^ cm^-1^. Protein concentration was measured using the Bradford assay (Pierce, Dallas, TX) adjusting the standard curve for the final concentration of lysis buffer added along with the unknown samples. Specific catalase activity was expressed as μmole/min/mg protein.

### Superoxide dismutase activity assay

This assay employs hematoxylin which, above pH 7.0, generates superoxide (SO) and is in turn oxidized by the SO [38]. The result is a concentration-dependent increase in absorbance with a spectrum between 400 and 670 nm. Superoxide dismutase (SOD) inhibits this increase in absorbance in a concentration dependent manner, and the enzyme activity is expressed as the percent of inhibition of the reaction without SOD.

Hematoxylin was dissolved in 0.05 M mono-potassium phosphate buffer pH 7.6 and stored in aliquots frozen at -80°C to ensure assay consistency. The reaction was started by adding 30 μl of the hematoxylin solution to 970 μl of 0.05 M potassium phosphate buffer and reading the absorbance increase at 560 nm. Curcumin does not absorb at this wavelength. This ratio generates an absorbance of 0.066 per minute, which allows the sensitivity necessary to detect SOD quantitatively.

Cells were harvested, washed in SS buffer, and pellets of 1x10^7^ cell were frozen at -80°C. Freshly prepared cold lysis buffer (0.05 M potassium phosphate buffer pH 7.6, 0.1% Triton X-100, and 1x protease inhibitor cocktail) was added to the frozen pellets and vortexed to lyse the cells. Dilutions were made with lysis buffer and samples of the dilutions were assayed for their ability to inhibit the reaction by the catalytic degradation of SO. The lysis buffer had no effect on the reaction. Protein concentration was measured as above.

#### Superoxide determination

Superoxide production was measured utilizing an assay based on the reduction of the tetrazolium dye XTT (2,3-Bis- (2-Methoxy-4-Nitro-5-Sulfophenyl)- 2H-Tetrazolium-5-Carboxanilide (Molecular Probes, Eugene, OR)) [39]. Stock solutions (10 mM) were prepared in 20 mM potassium phosphate buffer, pH 7.0. XTT was added to growing cells (2–4×10^6^ cells/ml) at a final concentration of 500 μM and incubated for the indicated times. Reaction with superoxide converts the pale yellow dye to a bright orange colored water soluble formazan product which can be measured by spectrophotometry at 470 nm [40, 41]. One ml aliquots of treated cells were centrifuged and the supernatant was measured in a spectrophotometer. Superoxide production was expressed as absorbance at 470 nm per 1x10^8^ cells.

### Hydrogen peroxide determination

Hydrogen peroxide (H_2_O_2_) was detected using an assay based on Amplex Red oxidation (Molecular Probes, Eugene, OR). In the presence of horseradish peroxidase (HRP) Amplex Red produces a red oxidation product, resorufin, upon reaction with H_2_O_2_ which is measured by spectrophotometry at 540 nm. Stock solutions were prepared according to the manufacturer’s instructions. A working solution of 100 μM Amplex Red reagent was prepared by mixing 100 μl of the supplied Amplex Red stock solution, 200 μl HRP stock and 9.7 ml HL5 medium. Fifty microliters of the Amplex Red working solution were transferred to microplate wells.

Cells were grown to 1–2×10^6^ cells/ml. Curcumin or vehicle was added to the cell cultures so that the concentrations were twice the desired final concentration. Immediately, 50 μl of the cells containing curcumin or vehicle were added to microplate wells containing 50 μl of Amplex Red working solution to bring final curcumin concentration to 1X. The plates were shaken at 200 RPM at 22°C and read at the indicated time points to measure H_2_O_2_ accumulation.

### qRT-PCR

Total RNA was prepared from 5×10^6^ cells 24 hours after addition of curcumin or vehicle using a RNEasy mini kit (Qiagen, Hilde, Germany). RT-PCR was performed using the High Capacity cDNA Reverse Transcription kit (Applied Biosystems (Foster City, CA)) following the manufacturer’s instructions. A cocktail of random primers (included with kit) was used to amplify the first strand cDNA, which then served as template for subsequent PCR. The *Ig7* gene was used as an internal control [42]. The oligonucleotide sequences are listed in S2 Table.

### Transcriptome analysis

To determine the global transcriptional response to increasing concentrations of curcumin, wild-type cells (2×10^6^ cells/ml) were grown in 20 ml of shaking culture in the presence of the indicated concentration of curcumin. Fifteen ml of the cultures were harvested at the indicated times. Total RNA was isolated using Ambion Trizol Reagent (Life Technologies, Carlsbad, CA) according to the manufacturer’s instructions. mRNAs were prepared by double poly(A) selection using a Dynabeads mRNA purification kit (Life Technologies, Carlsbad, CA), fragmented and reverse transcribed to short cDNAs [43]. Multiplexed cDNA libraries were prepared and sequenced using the Illumina sequencing platform as described [44]. The resulting sequences were aligned to the *Dictyostelium* reference genome using pipelines of Genialis GenBoard (Genialis, Houston, TX). The data were deposited in Gene Expression Omnibus (GEO; accession number, GSE97392). The reproducibility of biological replicates was tested using Spearman’s correlation between the transcriptomes as a quality control, and two of the RNA-seq data points were eliminated (Spearman’s correlation < 0.95) from subsequent analyses. For the visualization of relative distances between the transcriptomes, hierarchical clustering analysis (R package ‘hclust’) and multidimensional scaling (R function cmdscale) were performed [45, 46]. The differential expression analyses were performed two ways; (1) baySeq method using Genialis GenBoard (false discovery rates < 0.01, likelihoods > 0.9), and (2) calculating fold change using log_2_ of the ratio between samples. For GO term enrichment analyses on all gene sets, custom R scripts (R package ‘topGO’ version 2.14.0) were used [46].

## Results

### Curcumin reduces cell proliferation and viability

**Fig 1:**
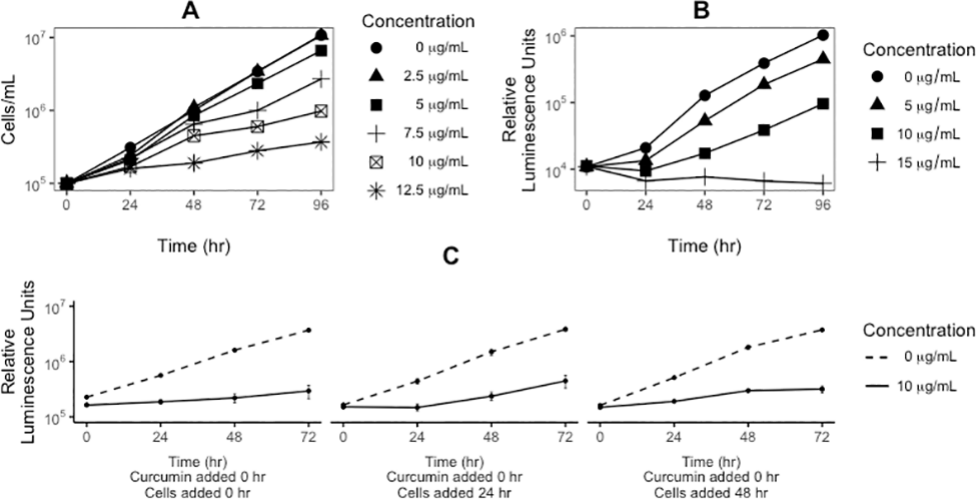
Curcumin reduces proliferation and cell viability. A) Axenically growing AX4 cells were treated with curcumin at the indicated concentrations and cell density was monitored over four days by direct counting with a hemocytometer. B) In separate experiments, viability of curcumin treated cells was assayed by measuring ATP in metabolically active cells using CellTiter-Glo® which measures cell viability. C) Curcumin stability in HL5 growth medium was determined by adding curcumin to medium at the onset of the experiment (0 hours). Flasks were inoculated with cells at time 0, 24 and 48 hours, and each assayed for 72 hours using the CellTiter- Glo® method. Taken together, these results show that curcumin has a lasting inhibitory effect on cell proliferation. Error bars in all figures represent the standard deviation compared to the mean.

We determined the effects of curcumin on *D*. *discoideum* cell proliferation by counting cells in the treated cultures. Fig 1A shows the results of treating axenically growing wild-type AX4 cells with different concentrations of curcumin over a period of 4 days. The data show that curcumin inhibits the rate of cell proliferation in a dose dependent manner where 2.5 μg/ml has almost no effect compared to the control and 12.5 μg/ml strongly inhibits cell proliferation.

We previously described a rapid and sensitive cell viability assay for *D*. *discoideum* using CellTiter-Glo® that measures ATP in living cells [36]. Using this assay the experiment in Fig 1A was repeated including a higher curcumin concentrations (15 μg/ml), and the data on rate of cell proliferation (Fig 1B) are virtually identical to those obtained by direct cell counting. Notably, 15 μg/ml curcumin inhibits all cell proliferation over 4 days. Taken together, the data in Fig 1A and 1B indicate that curcumin has a lasting inhibitory effect on cell proliferation over 4 days—that is, the rate of proliferation remains repressed.

Based on other reports that curcumin was unstable in aqueous solution we examined the stability of curcumin in the growth medium. Multiple flasks of medium containing 10 μg/ml curcumin were inoculated at daily intervals with cells at an initial density of 1 x 10^5^ cells/ml. As seen in Fig 1C the curcumin had the same inhibitory effect on cell proliferation irrespective of how long it was in the growth medium. Although this does not rule out that some breakdown product or derivative is responsible for the effect on growth, it does demonstrate that the effect does not diminish with the time curcumin is in the medium before the addition of the cells. Much more detailed chemical analysis would be needed to determine if curcumin is directly acting on the cells.

### Curcumin reduces catalase A and SOD enzyme activities

The effects of curcumin have been often attributed to antioxidant properties [22, 47, 48]. The levels of anti-oxidant enzymes such as catalase and superoxide dismutase are often used as indicators (surrogates) for the level of ROS in cells [49, 50]. Therefore, we measured the effect of curcumin on these enzymes.

**Fig 2:**
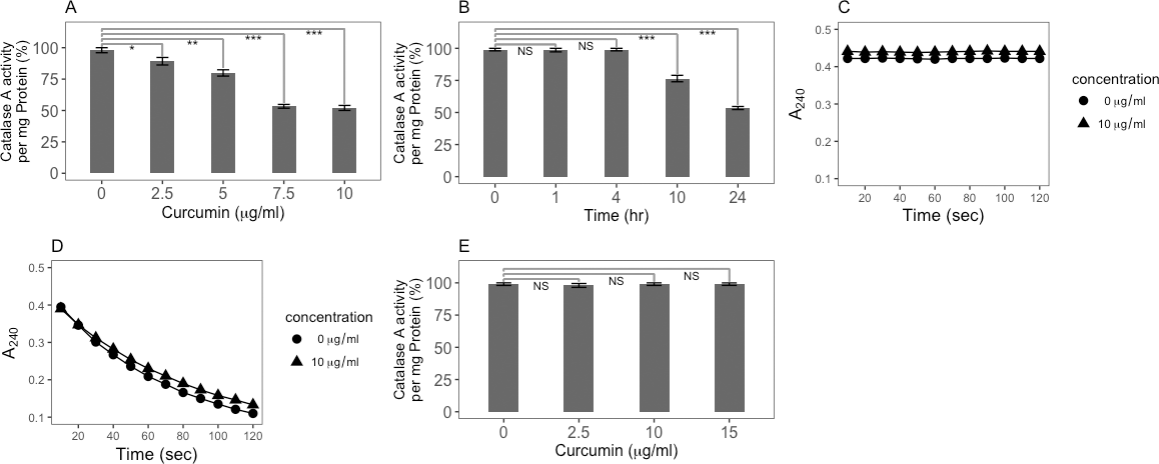
Curcumin reduces catalase A enzyme levels in wild-type cells. A) Catalase A enzyme specific activity is reduced in curcumin treated cells in a dose dependent manner; B) Reduction in catalase A specific activity is not immediate and takes up to 24 hours to manifest itself, indicating that it is not due to enzyme inhibition; C) Curcumin by itself does not have an effect on the stability of H_2_0_2_; D) Curcumin by itself does not have a direct effect on the rate of catalase A activity in cell extracts (5 μl of an extract of 2 x 10^7^ cells in 1 ml lysis buffer); and E) Curcumin by itself does not have an effect on the extent of the *in vitro* catalase A activity (5 μl of an extract of 2 x 10^7^ cells in 1 ml lysis buffer). Error bars represent the standard deviation of the mean. Statistical analyses were carried out using a two-tailed t-test. *p<0.05, **p<0.01, ***p<0.001.

Cells treated with curcumin were assayed for the level of catalase A enzyme activity. Catalase A is the only catalase enzyme in proliferating *D*. *discoideum* cells [37], although a second enzyme–catalase B–is expressed at a late stage of multicellular development [51]. The data demonstrate that the specific activity of catalase A in the presence of curcumin decreases to 50% in a dose (Fig 2A) and time (Fig 2B) dependent manner that is consistent with the inhibition of cell proliferation (Fig 1). Further *in vitro* experiments showed that this loss of catalase A specific activity in the presence of curcumin was not due to a direct effect of curcumin on the catalase A enzyme. Curcumin alone had no effect on the stability of H_2_O_2_ (Fig 2C), and neither the kinetics (Fig 2D) nor the extent (Fig 2E) of catalase A catalysis was effected by curcumin. These experiments (Fig 2C–2E) show that the effect of curcumin on catalase A activity is not immediate and not at the level of enzyme inhibition.

**Fig 3:**
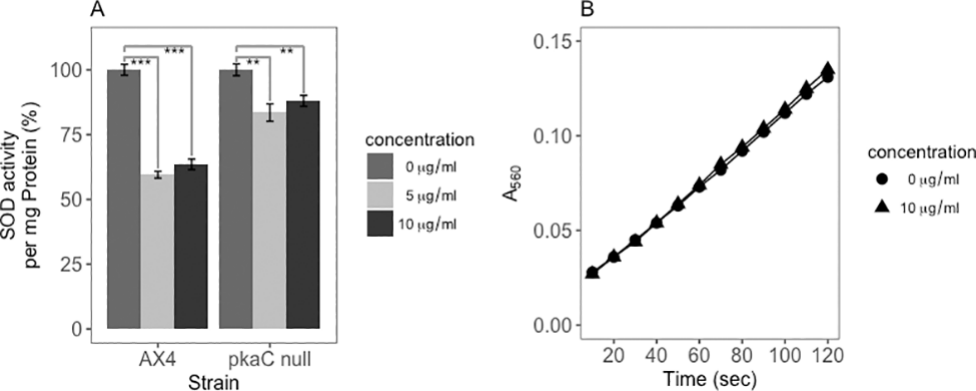
Curcumin reduces SOD enzyme activity in wild-type cells but not in *pkaC* null cells. A) SOD enzyme activity in curcumin treated parental AX4 cells (5 μg/ml and 10 μg/ml) is reduced by nearly half relative to untreated cells. In contrast, there is much less effect on SOD enzyme activity in *pkaC* null cells treated with curcumin for 24 hours. B) Curcumin has no effect on the rate of generation of superoxide in the assay. P-values are defined in Fig 2.

Superoxide dismutase (SOD) was also assayed in cells treated with curcumin. The assay relies on the inhibition of superoxide accumulation by the action of SOD. Fig 3A shows that, similar to the catalase A activity, there is a reduction in SOD activity after exposure to curcumin for 24 hours. This response was seen for both 5 and 10 μg/ml curcumin, although 5μg/ml had more effect on SOD activity than it did on catalase A activity (40% reduction vs. 20% reduction (Fig 2) and on cell proliferation. Moreover, there is no direct effect of curcumin on the generation of superoxide in this assay (Fig 3B). In contrast, the SOD enzyme activity in *pka*C null mutant cells (Fig 3A) is much less affected by curcumin (p<0.001 comparing 5 and 10μg/ml samples between strains) and the results will be discussed in greater detail below.

Taken together, these studies show that curcumin reduces the catalase A and SOD enzyme activities in wild-type cells, and suggests that curcumin is not acting as an anti-oxidant in *D*. *discoideum* cells.

### Curcumin negatively regulates antioxidant enzyme mRNA levels

**Fig 4:**
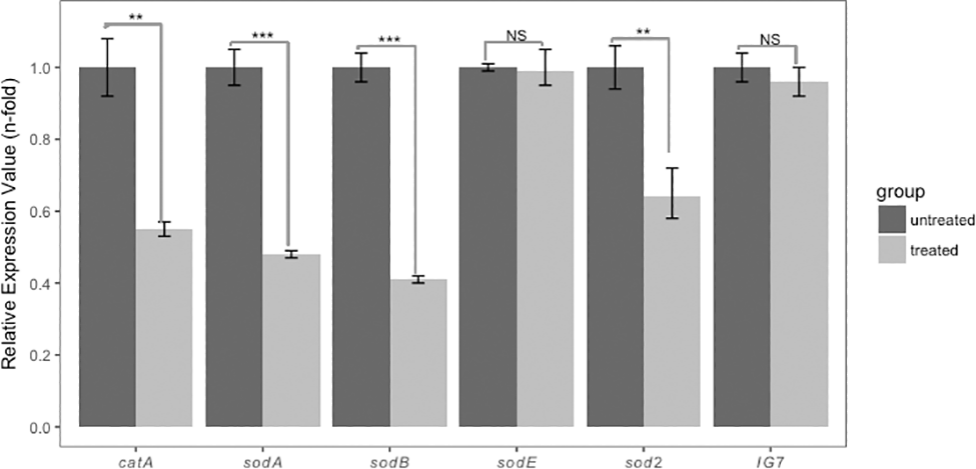
Curcumin negatively regulates antioxidant enzyme RNA levels. Total RNA was prepared from 5×10^6^ axenically growing cells treated with 10 μg/ml curcumin for 24 hours. Transcript levels of the antioxidant enzymes, *catA*, *sodA*, *sodB* and *sod2* are reduced in cells treated with curcumin. P-values are defined in Fig 2.

Based on the results of the catalase A and superoxide dismutase enzyme assays we wanted to determine if the decrease in enzyme specific activities was due to a change in mRNA level. Thus, we measured the mRNA levels of catalase A (*catA*) and superoxide dismutase (*sod*) A, B, E and 2 in curcumin treated cells. The data in Fig 4 show that *catA* mRNA is reduced by 50% in curcumin treated cells. In similar fashion *sodA*, *sodB* and *sod2* mRNA were all reduced in response to the curcumin treatment. In contrast, *sodE* mRNA was unchanged in curcumin treated cells. Taken together, these results suggest that curcumin negatively regulates the mRNA levels for 4 antioxidant enzymes, and is consistent with the down-regulation of catalase A and SOD enzyme activity levels in curcumin treated cells. The data indicate that curcumin is negatively regulating the transcription of these anti-oxidant enzyme genes.

### Curcumin upregulates ROS levels

**Fig 5:**
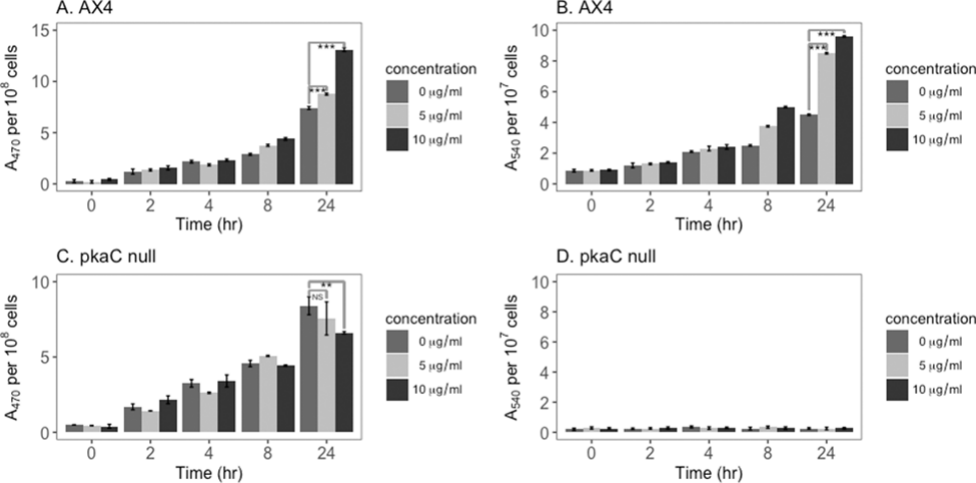
Curcumin up-regulates superoxide and H_2_O_2_ in wild-type cells but not in *pkaC* null cells. Logarithmically growing wild-type AX4 (A and B) or *pkaC* null cells (C and D) were treated with curcumin at the indicated concentrations, and superoxide (A and C) or H_2_O_2_ (B and D) levels were determined as described in Material and Methods. The results indicate that curcumin increases the level of superoxide and H_2_O_2_ in a dose-dependent manner in AX4 cells but not in the *pkaC* null cells. P-values are defined in Fig 2.

The data demonstrating that curcumin lowered the levels of the catalase and SOD enzymes (Figs 2 and 3) and mRNAs (Fig 4) suggested that this would result in an increase in ROS. Thus, we measured the levels of superoxide and H_2_O_2_ in cells. Developing *D*. *discoideum* cells produce superoxide that can be measured by the oxidation of XTT [41]. Fig 5A shows that proliferating AX4 cells also produce superoxide, and that curcumin treatment causes an increase in the level of superoxide produced in a dose dependent fashion (p<0.001 comparing 5 and10 μg/ml at 24 hours). Both 5 and 10 μg/ml curcumin increase superoxide production over the untreated control showing a statistically significant increase at 24 hours. Therefore, proliferating cells produce superoxide, and treatment with curcumin results in increased production of superoxide with kinetics that resemble the kinetics of the decrease in the superoxide dismutase enzyme activity and mRNAs.

The effect of curcumin on the level of H_2_O_2_ in AX4 wild-type cells was also determined. Fig 5B shows dose- and time-dependent increases in H_2_O_2_ in wild-type cells treated with curcumin (p valuses <0.001). The pattern of H_2_O_2_ accumulation in curcumin treated cells also parallels the kinetics of decrease in the level of catalase in the presence of curcumin. Thus, both H_2_O_2_ and superoxide increase in curcumin treated AX4 cells as predicted by the decrease in the levels of the enzymes that degrade these reactive oxygen species, further suggesting that curcumin is not acting as an anti-oxidant. Parallel analyses on *pkaC* null cells (Fig 5C (superoxide) and 5D (H_2_O_2_)) show no effect of curcumin on SO and H_2_O_2_ levels and are discussed in greater detail below. Remarkably, the *pkaC* null cells produce dramatically lower levels of H_2_O_2_ than the AX4 cells.

### Oxidants have a different effect on proliferating cells than curcumin

**Fig 6:**
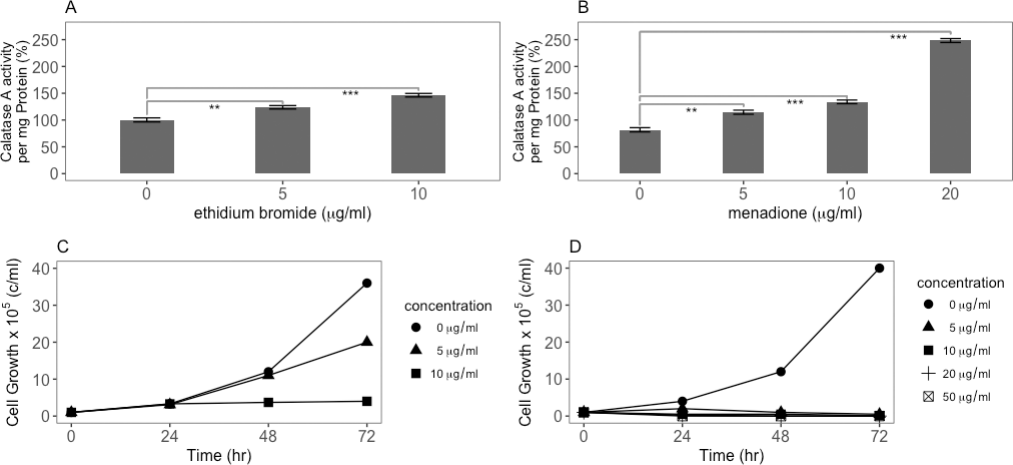
Oxidants cause an increase in catalase A enzyme activity. A) In contrast to the effect of curcumin, catalase A activity is increased in cells treated with ethidium bromide for 24 hours in a dose dependent manner. B) The same effect is observed with treatment with another oxidant, menadione. These results indicate that *D*. *discoideum* cells, like mammalian cells, respond to oxidative stress by upregulating antioxidant enzymes and that the effect curcumin has on catalase A enzyme specific activity (Fig 2A and 2B) is not the direct result of oxidative stress. C) Ethidium bromide at 5 and 10 μg/ml inhibits cell proliferation of parent cells. D) Menadione at 5, 10, 20 and 50 μg/ml inhibits proliferation of parental cells. P-values are defined in Fig 2.

Our findings suggest that curcumin is not acting as an antioxidant in *D*. *discoideum* cells. Previous studies suggest that curcumin can act as a pro-oxidant in some situations [25, 26, 52]. To this end we tested the effects of two pro-oxidants, ethidium bromide and menadione, on proliferating wild-type cells. Treatment with either pro-oxidants resulted in an increase in catalase specific activity (Fig 6A and 6B). This is in contrast to the decrease in catalase enzyme specific activity in the presence of curcumin. Thus, like mammalian cells, *D*. *discoideum* appears to respond to oxidative stress by upregulating antioxidant enzymes [53]. Fig 6C and 6D show that both ethidium bromide and menadione have an inhibitory effect on cell proliferation. This result indicates that the increased generation of ROS by curcumin is not the cause of the reduction in the antioxidant enzyme RNAs and enzymes. Rather it suggests that the increase in ROS is due to the reduction of antioxidant enzyme production.

### The anti-oxidant N-acetylcystiene affects cells differently than curcumin and does not reverse the effects of curcumin

**Fig 7:**
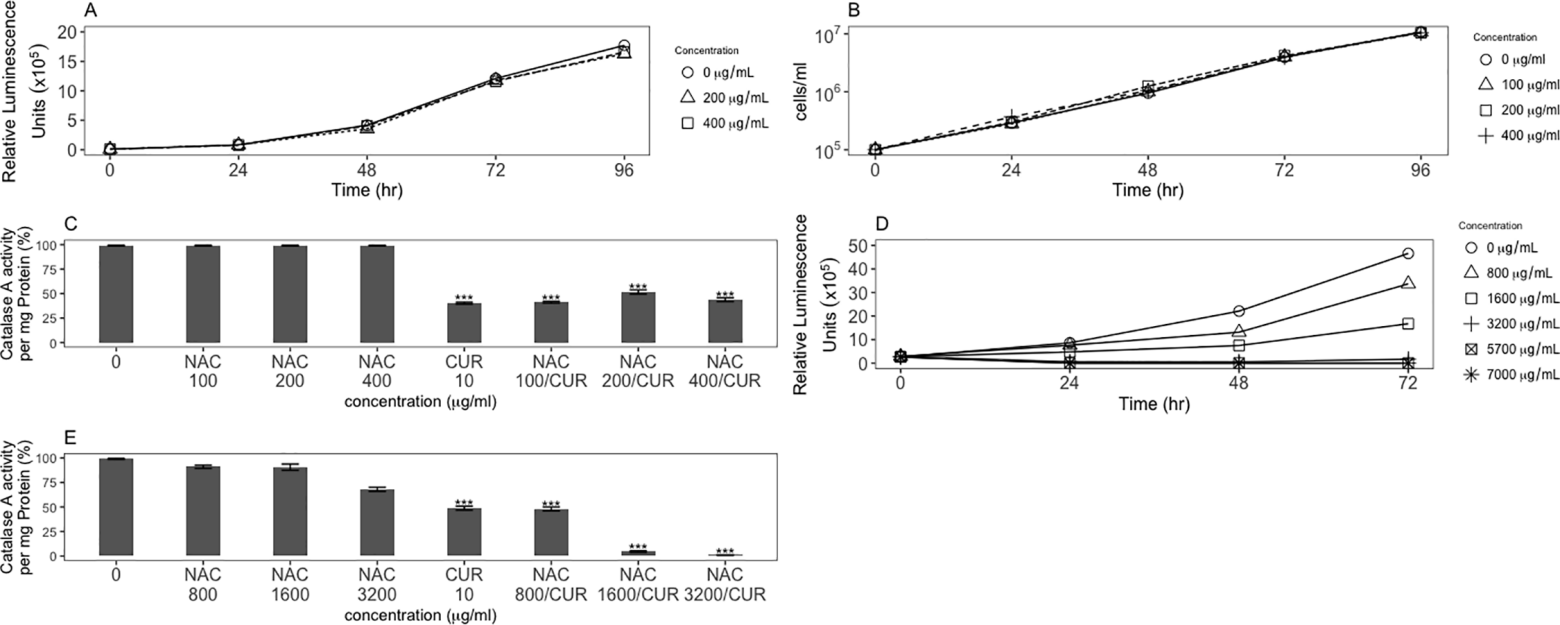
The antioxidant NAC affects cells differently than curcumin and does not reverse the oxidant effect of curcumin. The antioxidant NAC, known to counter the effect of oxidative stress, does not have any effect on cell proliferation of wild-type *D*. *discoideum* cells: A) Cell Titer Glo assay and B) direct cell counting. C) NAC did not counter the effect of curcumin on cells treated for 24 hours, indicating that the effect of curcumin on catalase A specific enzyme activity was not directly due to oxidative stress. D) Increased NAC concentrations inhibit cell proliferation at very high concentrations. E) However, these increasing concentrations of NAC still do not counter the effect of cells treated with curcumin for 24 hours. P-values are defined in Fig 2.

Based on the previous demonstration that oxidants affected catalase levels in *D*. *discoideum* cells differently than curcumin, it was of interest to examine the effect of the antioxidant N-acetylcystiene (NAC). NAC at 2 mM (326 μg/ml) inhibits chemically induced production of ROS in human SH-SY5Y neuroblastoma cells [54]. NAC treatment (between 100 and 400 μg/ml) had no effect on cell proliferation in *D*. *discoideum* (Fig 7A and 7B). Addition of NAC did not reverse the reduction of catalase A specific activity by curcumin at any of the doses tested (Fig 7C). Our previous studies showed that NAC could inhibit the terminal stages of multicellular development, but required concentrations 15-fold higher for this effect [51]. To test whether the concentrations of NAC used in Fig 7A and 7B were not high enough to elicit an antioxidant response, NAC concentrations were increased up to 7000 μg/ml. Indeed, at these higher concentrations, NAC does have an effect on cell proliferation (Fig 7D). However, even at these concentrations NAC does not reverse the decrease in the level of catalase A enzyme activity caused by curcumin treatment (Fig 7E), supporting the idea that curcumin is not acting directly as an oxidant (see Fig 6). However, it is possible that NAC does not have an effect on *D*. *discoideum* cells except at high concentrations.

### PKA is involved in the cellular response to curcumin

**Fig 8:**
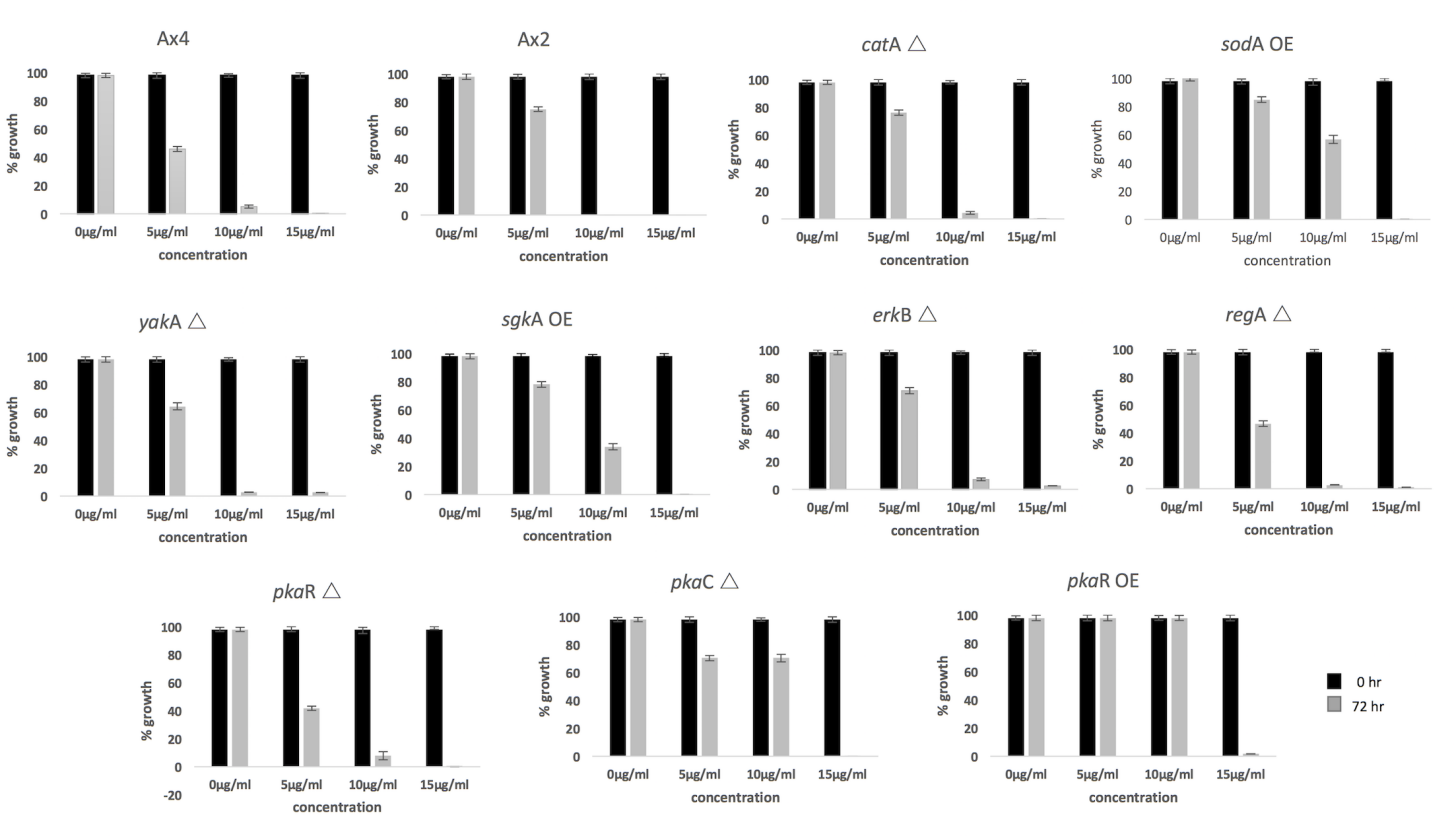
Examination of candidate genes reveals that PKA is required for the cellular response to curcumin. The indicated mutants, described in detail in the text, were tested for their sensitivity to curcumin over time. Each mutant was tested multiple times and in parallel with the wild-type AX4 strain. Cell proliferation was measured using the CellTiter-Glo® assay, and expressed as percent of the control untreated samples at 72 hours. Δ = null mutant, OE = over-expressing mutant.

A variety of existing mutant strains with deleted or over-expressed genes in known regulatory/signaling pathways or genes involved in generating or scavenging ROS were tested for their response to curcumin (S1 Table). The results are shown in Fig 8.

Wild-type strains AX4 and AX2. The parental AX4 cells show a dose dependent inhibition of cell proliferation by curcumin with essentially complete inhibition of proliferation at 10 μg/ml curcumin. Similar results were obtained with AX2.

Antioxidant mutants *catA* null and *sodA* over-expressor. The *catA* gene is expressed uniformly in mitotically dividing cells and throughout development [55]. It has been studied extensively in terms of its role in resistance to methanol and unrelated compounds such as thiabendazole, and its role in resistance to hydrogen peroxide and other cellular stresses [37, 51]. Based on the finding in Fig 2, it was possible that cells lacking *catA* (strain IR41; AX4 background) would have an altered response to curcumin. However, the resistance of the *catA* null cells to curcumin is identical to that of the parent strain. Thus, *catA* does not appear to be a primary defense against the effects of curcumin.

*sodA* is one of seven superoxide dismutase genes in *D*. *discoideum*. The *sodA* over-expressing strain (AX2 background) was characterized and shown by western analysis to produce an increase in SODA protein compared to the parent strain [41]. *sodA* was one of the genes shown in Fig 4 to be down-regulated after exposure to curcumin, and we have also documented in Fig 5 that superoxide (SO) is up-regulated in response to curcumin in a temporally correlated way to the loss of SOD. Examination of the SOD over-expressor mutant showed an increase in resistance to curcumin in the presence of 10 μg/ml curcumin in contrast to the parent cells.

*yakA* null cells. *yakA* encodes a protein kinase which is required for the growth to development transition in *D*. *discoideum* [56] and has been implicated in the modulation of stress response in *D*. *discoideum* [57]. In that study, *yakA* mutant cells (AX4 background) were shown to have increased sensitivity to nitrosoactive/oxidative stress relative to the parent strain. This suggested that there might be a relationship of *yakA* to curcumin sensitivity based on the increase in ROS in curcumin treated cells (Figs 5 and 6). However, *yakA* null mutants show similar sensitivity to curcumin as the parent cells.

Sphingosine kinase A over-expressor mutant. A number of studies have reported that bioactive sphingolipids are involved in the cellular response to curcumin in a variety of mammalian cell types [58–60]. Sphingolipids have been studied in *D*. *discoideum*, and the sphingosine kinase A over-expressing strain (SA604; AX4 background) was shown to have increased resistance to the chemotherapeutic drug cisplatin [61]. These cells showed a modest increase in resistance to curcumin at 10 μg/ml.

Developmental regulatory *erkB* null mutant and *regA* null mutant. The *erkB* gene is a key regulator of development [62]. ERK-B activates adenylyl cyclase as well as inhibiting the REG-A phosphodiesterase [63]. This mutant (AK240; AX4 background showed similar sensitivity to curcumin as the parent cells.

*regA* encodes a phosphodiesterase [64] that negatively regulates the activity of protein kinase A (PKA) by catalyzing the hydrolysis of the PKA activator cAMP [65]. Interestingly, a random insertional mutagenesis study done in *D*. *discoideum* to investigate the molecular mechanisms underlying resistance to the anti-cancer drug cisplatin, identified *regA* as one of the genes involved in the cellular response to cisplatin [66]. However, the *regA* null mutant (HM332; AX4) showed a similar dose dependent effect of curcumin on cell proliferation as seen with the parent cells.

Protein kinase A (*pkaR* null, *pkaR* over-expressor and *pkaC* null mutant). The cAMP-dependent protein kinase A is known to regulate the activity of a wide number of proteins. This well characterized enzyme in *D*. *discoideum* plays a central role in development affecting aggregation, chemotaxis, and spore differentiation [63, 67], and has important regulatory functions in human, zebrafish, Drosophila, yeast, and *D*. *discoideum*. *D*. *discoideum* PKA is made up of a single regulatory subunit (PKA-R) and a single catalytic subunit (PKA-C) [68, 69]. cAMP binding to PKA-R causes the dissociation of PKA*-*C rendering PKA-C active. A mutant strain, *pkaR*Δ (AX4 background), harboring a null mutation in the regulatory subunit showed similar sensitivity to curcumin as the parent. However, two strains, one carrying a null mutation in *pkaC* (AX4 background) and another over-expressing the regulatory subunit *pkaR* (4M; AX2 background) both showed greatly increased resistance to curcumin at 10 μg/ml compared to the parent strain. Both the *pkaC* null mutant and the *pkaR* over-expressing mutant have normal growth rates (the same as wild-type cells) indicating that increased resistance to curcumin is not due to a defect in proliferation capacity *per se*.

These analyses indicate that protein kinase A is an important regulator of the cellular response to curcumin. In addition, *sodA* and to a lesser extent sphingosine kinase A also play a role in the response. We chose to focus on the role of protein kinase A.

**Fig 9:**
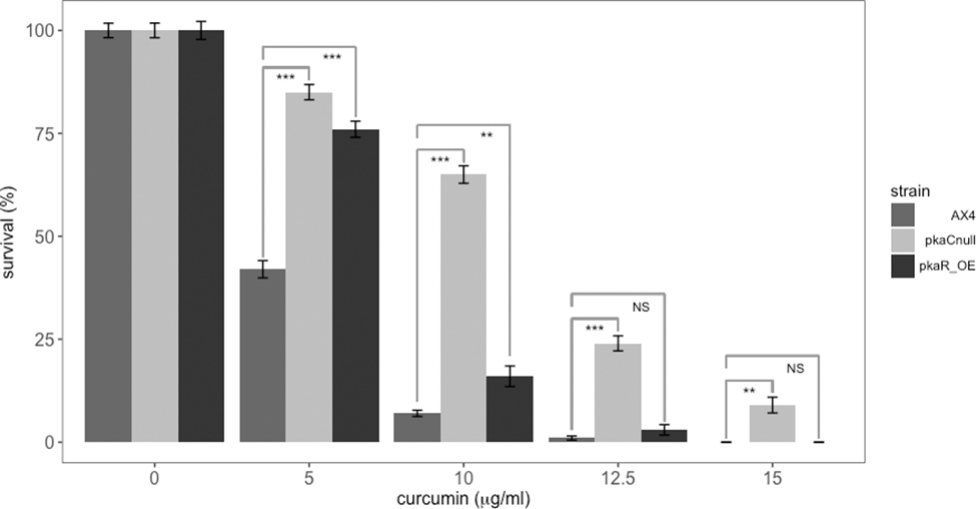
Validation that the *pkaC* null and *pkaR*-OE mutants are more resistant to curcumin. Wild-type, *pkaC* null and *pkaR*-OE cells were clonally plated in association with *Klebsiella aerogenes* on SM plates containing curcumin at the indicated concentrations. Plaques resulting from clonal growth were counted and the percent survival was calculated. The results are from multiple platings within the linear range of the assay. The data confirm that the *pkaC* null and *pkaR*-OE cells are more resistant to curcumin than the wild-type cells. P-values are defined in Fig 2.

To further examine the relationship between the curcumin response and PKA, we performed a different viability assay by clonally plating these mutant strains in association with *Klebsiella aerogenes* in the presence of curcumin. Results from this experiment confirmed that cells with an inactive form of PKA (*pkaC* null or *pkaR*-OE) showed increased resistance to curcumin than the parent strain (Fig 9).

### The catalase A and SOD enzyme activities are not affected by curcumin in *pkaC* null cells

**Fig 10:**
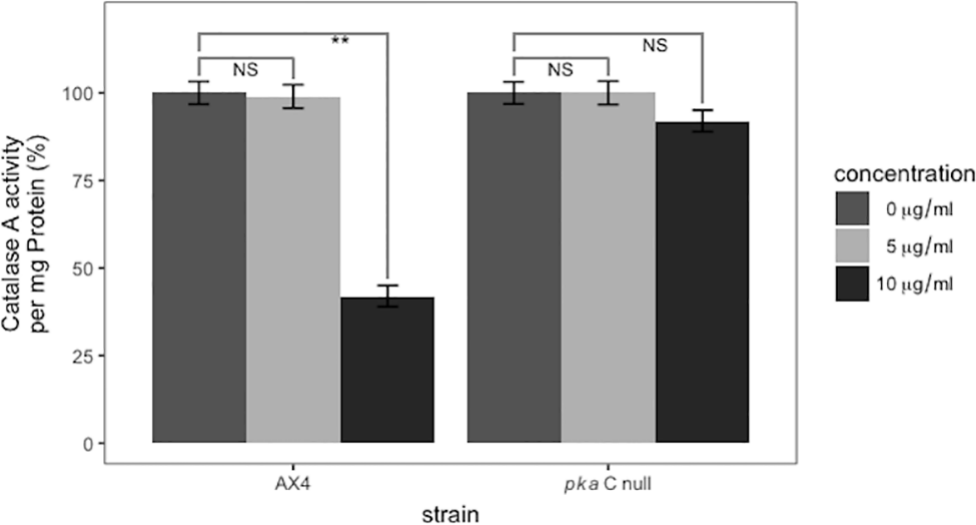
Catalase A enzyme levels are unchanged in curcumin treated *pkaC* null cells. Unlike wild-type cells, *pkaC* null cells treated with curcumin for 24 hours did not show a change in catalase A activity/mg protein enzyme activity. P-values are defined in Fig 2.

*pkaC* null cells treated with curcumin were assayed for the level of catalase A enzyme activity. The data demonstrates that concentrations up to 10 μg/ml of curcumin did not affect catalase A enzyme activity in *pkaC* null cells (Fig 10), in contrast to the decrease in *catA* specific activity in the parental cells, confirming previously seen results where the level of catalase A enzyme activity in AX4 cells was reduced (Fig 2).

*pkaC* null cells treated with curcumin for 24 hours showed only a small decrease in total SOD enzyme activity compared to the treated wild-type cells (Fig 3A). The data support the idea that the SOD enzymes, like the catalase A enzyme, are not all down-regulated by curcumin in the *pkaC* null cells.

### ROS levels in *pkaC* null cells are not upregulated by curcumin

Previous data had shown that catalase A and SOD enzyme specific activities in AX4 cells decrease upon treatment with curcumin (Figs 2 and 3), resulting in an increase in SO and H_2_O_2_ levels (Fig 5A and 5B). Curcumin had little to no effect on catalase A and SOD activity in *pkaC* null cells (Figs 10 and 3A), suggesting that ROS may not be up-regulated as observed with the wild-type strain. Thus, we tested the *pkaC* null cells for the effect of curcumin on SO production. As shown in Fig 5C curcumin does not increase the level of SO being generated in the *pkaC* null cells. Indeed, the curcumin treated cells have less SO at 24 hours than the untreated cells. These results are in agreement with the relative lack of SOD enzyme down-regulation observed in curcumin treated *pkaC* null cells (Fig 3A). In similar fashion, as seen in Fig 5D, the level of H_2_O_2_ does not increase in the curcumin treated *pkaC* null cells compared to the parental AX4 cells (Fig 5B). Indeed, this mutant shows virtually no increase in H_2_O_2_ level in the presence or absence of curcumin indicating a striking difference in cell physiology in this mutant and suggests that *pka* is involved in some way to the generation of H_2_O_2_. Thus, *pkaC* null cells do not respond to curcumin by up-regulating the level of ROS, presumably as a result of not down-regulating their catalase A and SOD enzyme levels.

### Transcriptome analysis of the effects of curcumin treatment

The down-regulation of SOD and catalase demonstrated that curcumin had an effect on transcription, so we determined what other genes had their transcription altered by curcumin. We treated cells with curcumin at multiple concentrations (0, 2.5, 5.0, 7.5 and 10.0 μg/ml) and sampled them at multiple time points (0, 4, 8 and 12 hour).

Triplicate samples of were prepared for the RNA-seq experiment. Equivalent RNA was extracted from each sample. Each transcriptome was analyzed for consistency between biological replicates (S1 Fig) In all cases, the biological replicates exhibited high reproducibility with the exception of two samples (2.5 μg/ml at 12 hours and 7.5 μg/ml at 8 hours) that were eliminated from the following analyses. Examination of the correlation between any two samples showed that the differences in transcriptional profiles are relatively small compared to the massive changes in gene expression seen during the multicellular phase of development [70]. This observation implies that the effect of curcumin, while clearly affecting the transcription of multiple genes, is relatively nuanced.

**Fig 11:**
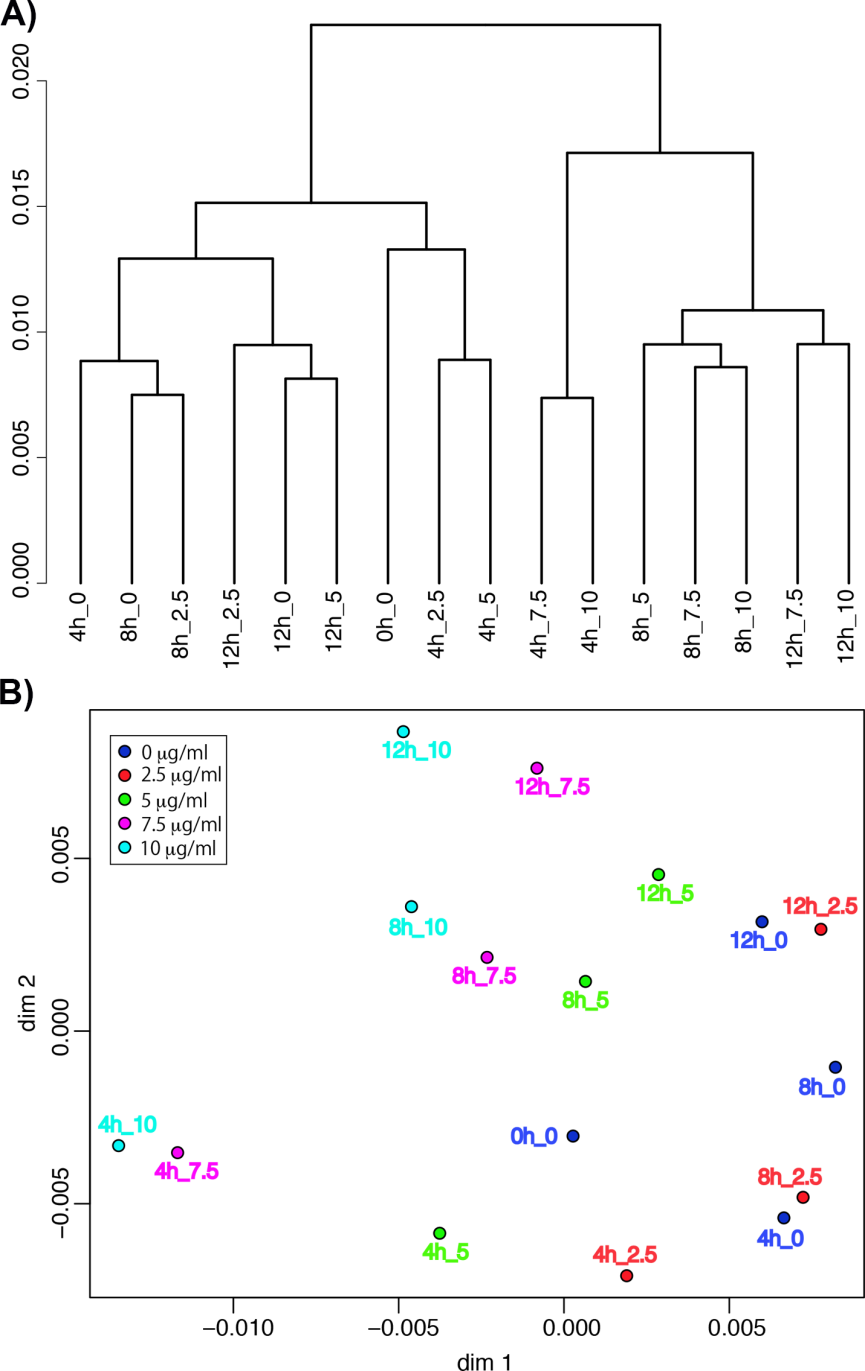
Transcriptome analysis of curcumin treated samples. We averaged the values of the replicate RNA-seq samples at each time point and each curcumin concentration as indicated, and calculated the distance (Spearman’s correlation) between the samples. A) Hierarchical clustering. The hierarchical clustering dendrogram illustrates the relationships between the samples based on these distance calculations. In the dendrogram, each leaf represents one condition (time and curcumin concentration) and the vertical distances between the joints represent the dissimilarity between the samples (see scale on the left, arbitrary units). B) Multidimensional scaling. Each point on the graph represents a sample (time and curcumin concentration, as indicated) and the distances between the points represent the dissimilarity between them–the closer two point are, the more similar they are. The axes units are arbitrary.

The underlying idea when comparing the transcriptional profiles of the samples by hierarchical clustering is to treat each profile as a molecular phenotype [71]. Each leaf of the dendrogram in Fig 11A represents transcriptional profiles averaged over biological replicates and the linkage pattern illustrates the relationships between the samples. The data show that there is a concentration effect on the transcriptome with a clear threshold between the 0, 2.5 and 5 μg/ml samples and the 7.5 and 10 μg/ml samples. These data support the cell proliferation phenotypes we observed in Fig 1, where there was little effect on cell proliferation at curcumin concentrations below 7.5 μg/ml. Fig 11A shows that that curcumin concentration is the primary factor in the transcriptional response.

Multidimensional Scaling (MDS) provides another method to examine the data [45]. The transcriptome of each strain can be thought of as a single point in a multidimensional space, where each gene defines a dimension and each mRNA abundance value determines the position of the point in that dimension. An MDS plot provides a two-dimensional view that is the best representation of the multidimensional distances between the points. When two points in the graph are close, it means that the transcriptomes of the two samples are similar to one another. Fig 11B shows that this analysis supports the same interpretation shown by hierarchical clustering analysis (Fig 11A) where there is a clear concentration threshold of the effect of curcumin on transcription in proliferating wild-type *D*. *discoideum* cells.

### Curcumin has an early transient effect on gene expression in *D*. *discoideum*

**Fig 12:**
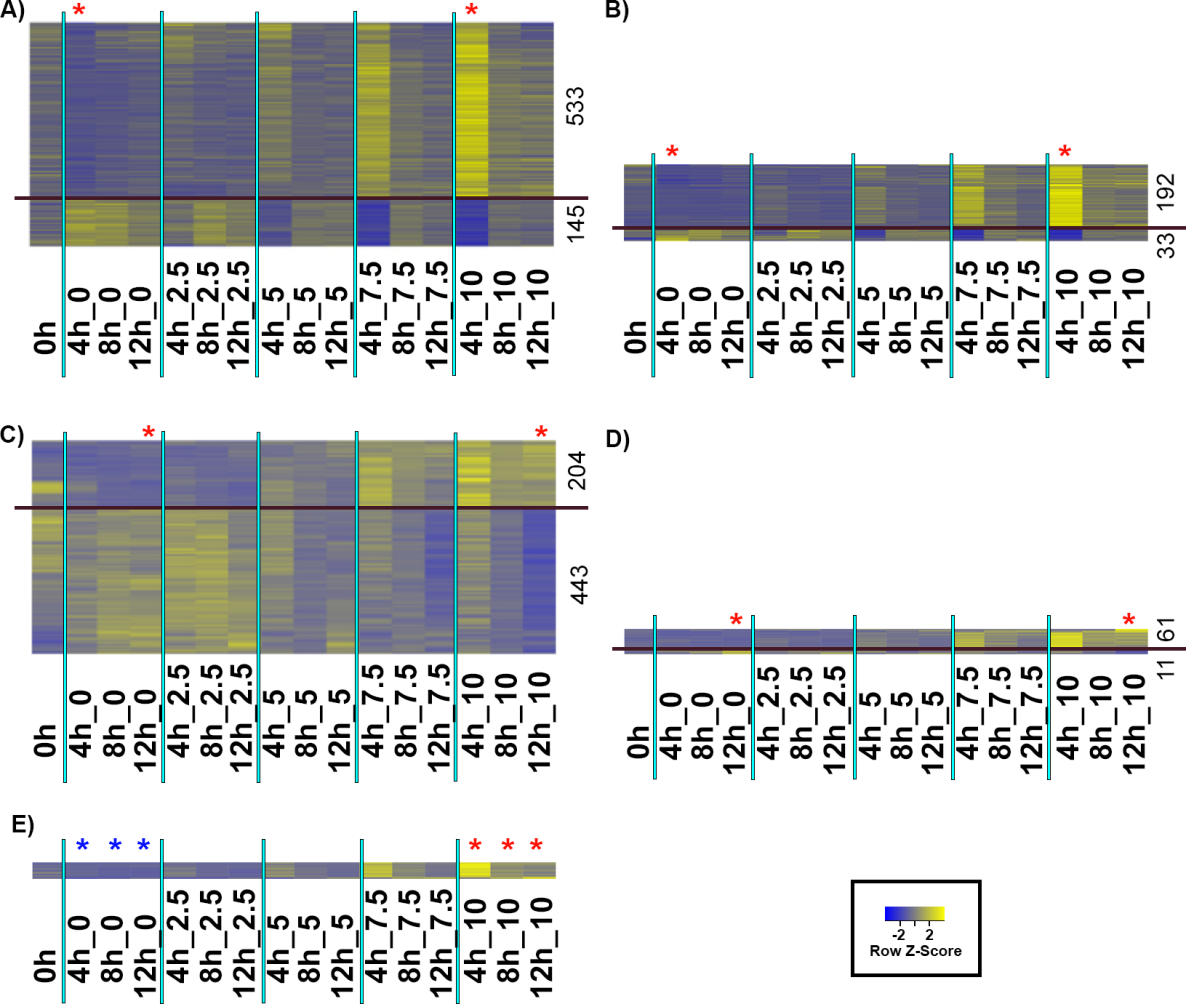
Heat map of differentially expressed genes following curcumin treatment. **A)** A differential expression analysis (using baySeq) of the 4 hour samples treated with 0 and 10 μg/ml curcumin (see two red asterisks). The yellow-blue heat map shows the differentially expressed genes at 4 hours. In the heat maps, each row represents the abundance levels of one transcript (scale indicated in the box) and each column represents one condition (time and curcumin concentration). Transcripts that exhibited increased abundance with increased curcumin concentration are clustered above the line (up-regulated), and transcripts that exhibited reduced abundance are clustered below the line (down-regulated). The number of genes in each cluster is indicated. B) Heat map showing differentially expressed genes with at least 3-fold change between 0 and 10 μg/ml curcumin at 4 hours. The above analyses in A) and B) were repeated for 0 and 10 μg/ml samples at 12 hours, C) and D), respectively. E) Heat map showing the expression patterns of the genes that are differentially expressed between untreated and treated (10 μg/ml curcumin) at all time points. Genes that were differentially expressed in the absence of curcumin were subtracted.

To identify early changes in gene expression following curcumin treatment, we compared the transcriptional profiles of cells treated with 0 and 10 μg/ml curcumin at 4 hours (Fig 12A and 12B). The baySeq differential expression analysis revealed that the transcription of 678 genes were effected– 533 genes were up-regulated and 145 were down-regulated in the treated cells (Fig 12A). The heat map of those genes in all samples shows that those effects are dose-dependent and transient and that these up-regulated mRNAs do not accumulate with time (Fig 12A). We also examined the expression pattern of the genes showing a minimum 3-fold change after curcumin treatment which limited the numbers to 192 up-regulated and 39 down-regulated genes (Fig 12B). This analysis shows the same early, transient transcriptional response as the previous analysis using the larger set of affected genes.

We performed Gene Ontology (GO) term enrichment analysis on the 533 genes up-regulated in the first 4 hours of treatment and found groups of genes encoding oxidoreductase enzymes, osmotic/salt/heat stress enzymes, ABC transporters and cell cycle related proteins (Table A in S3 Table). GO term enrichment analysis of the down-regulated genes provided an interesting counterpoint (Table B in S3 Table). The down-regulated groups include genes involved in apoptotic DNA fragmentation, defense from bacteria, and peroxisome function. It is notable that genes involved in sphingomyelin catabolism are also down-regulated, because of reports that sphingolipids are involved in regulating the cellular response to curcumin [58–60].

### The early effect on transcription is replaced with a unique transcriptional profile after extended exposure to curcumin

The differential expression analysis of the 4-hour time points indicated a robust and transient early transcriptional response to curcumin. To test whether there was a subsequent transcriptional response, we analyzed the 12-hour time points by comparing untreated cells to those treated with 10 μg/ml curcumin (Fig 12C and 12D). baySeq differential expression analysis of samples from the later time point exhibit more genes that are down-regulated (443) than are up-regulated (204) (Fig 12C). However, when we examined only those genes showing a 3-fold change, the number of down-regulated genes dropped to 11 (2.5%) whereas the up-regulated genes decreased to 61 (29.9%) (Fig 12D).

GO term enrichment analysis of the 12-hour samples (Table A in S4 Table) indicates that up-regulated genes encoding proteins involved in oxidoreductase activity, transcription factors, antioxidant activity, vitamin binding and the response to abiotic stimuli were affected by the prolonged curcumin treatment. Some of these genes (e.g., the oxidoreductase genes) are also up-regulated in the early 4-hour samples, but others are uniquely up-regulated at 12 hours. In contrast, the down-regulated genes of the 12-hour samples (Table B in S4 Table) encode proteins involved in cell cycle control, DNA replication, responses to drugs, and oxidoreductase activity. Overall, the GO term enrichment lists at 12 hours widely differ from those at 4 hours and confirm the analyses of the transcriptomes (Figs 11 and 12).

### Comparison between the 4 and 12-hour transcription profiles

To analyze the transcriptional response in greater detail, we compared the early (4-hour) and late (12-hour) effects of curcumin on the transcriptional profile of treated or untreated cells, respectively. The genes that exhibited differential expression between 4 and 12 hours in the untreated samples were subtracted from the genes that showed differential expression between the early and late time points in the treated populations. The results of GO analyses of the 67 up-regulated genes and 23 down-regulated genes in the absence of curcumin are shown in Table A in S5 Table and Table B in S5 Table, respectively. The up-regulated genes are predictably involved in various cell cycle associated functions. Table A in S6 Table and Table B in S6 Table list the genes that were up-regulated (161) and down-regulated (733) respectively after omitting the changes that were independent of curcumin treatment (S5 Table). Up-regulated genes include those involved in oxidoreductase activity, glutathione reductase activity, response to oxidative stress, and sphingolipid catabolic processes. In contrast, a number of cytochrome P450 related genes and DNA helicase and strand elongation genes are down-regulated.

We analyzed the transcription profiles between of untreated and treated cells at all time points (Fig 12E). Genes that were differentially expressed in the absence of curcumin were subtracted. This resulted in a smaller GO list of 50 genes that were up-regulated during curcumin treatment. The heat map of the genes show a rapid up-regulation at 4 hours and the levels gradually decline at later time points, confirming the time and dose dependency of the response to curcumin. These genes involved with oxidoreductase activity were highly enriched (S7 Table).

## Discussion

Botanicals are widely used as dietary supplements and for the prevention and treatment of disease. Despite a long history of use, there is generally little evidence supporting the efficacy and safety of these preparations. Curcumin has been used to treat a myriad of human diseases and is widely advertised and marketed for its ability to improve health [72]. The literature on curcumin does not provide a cohesive narrative that suggests how curcumin interacts with cells and affects cell physiology.

In this study we employed *Dictyostelium discoideum* as a lead system to examine the fundamental effects of curcumin on cells, and to begin to parse apart the underlying molecular mechanisms. Studies in *D*. *discoideum* have previously provided novel insights into the molecular mechanisms which cells use to respond to natural products and drugs widely used in human health care [30, 31, 33, 73], [28, 29]. The current work demonstrates that this system is equally useful for investigations into the fundamental physiological effects of botanicals.

The data demonstrate that curcumin or some breakdown product/derivative has a profound effect on cell proliferation and an unexpected and pleiotropic effect on gene transcription. Initial experiments on candidate antioxidant genes showed that three superoxide dismutase genes and the catalase A gene were down-regulated by curcumin, contrary to what was expected if curcumin was acting directly on the cells as an antioxidant. Curcumin also causes the cells to accumulate superoxide and H_2_O_2_. It is unlikely that the accumulation of superoxide and H_2_O_2_ is the proximal cause of the reduction in anti-oxidant enzyme mRNA levels because neither the pro-oxidants ethidium bromide and menadione nor the anti-oxidant NAC had the same effect on the level of catalase A activity as curcumin. The conclusion from these observations is that curcumin works through a non-redox mechanism to control mRNA levels of the genes encoding the anti-oxidant catalase A and SOD enzymes, and that the reduction of the enzymes in turn causes the accumulation of ROS (Fig 5).

There was virtually no knowledge of the function of the superoxide dismutases in *D*. *discoideum* despite their involvement in human disease including amyotrophic lateral sclerosis [74]. The current studies show that they may play a role in the response of cells to xenobiotics in the environment. The antioxidant genes are down-regulated by curcumin and conversely cells over-expressing SOD are more resistant to curcumin (Fig 8). It will be important in the future to construct a library of mutants with each of the SOD genes individually deleted so that their specific involvement in the response to xenobiotics can be accessed. Catalase A has been more extensively studied in *D*. *discoideum*. Both catalase A and SOD enzyme levels are down-regulated by curcumin. Previously, it was shown that transcription of the catalase A gene was not affected by exogenous H_2_O_2_ [37].

There are a number of paths to investigate the underlying mechanisms regulating curcumin sensitivity that take advantage of the genetic tractability in *D*. *discoideum*. These include 1) a candidate gene approach using the now extensive library of isogenic mutant strains that each differ from the parent by one gene (see Dictybase.org); 2) insertional mutagenesis [75] and chemical mutagenesis followed by whole-genome sequencing [76] to randomly inactivate genes in cells which can subsequently be identified by virtue of the cells’ increased resistance to curcumin; and 3) a gene expression strategy such as microarrays or RNAseq [70] to identify alteration of the expression patterns of genes and gene networks in response to curcumin. All of these approaches have been successfully employed in *D*. *discoideum* to investigate problems in cell and developmental biology including studies of importance to human diseases such as cancer and chemotherapy [33], and two of them have been used in this study.

The analysis of defined mutants demonstrated that protein kinase A mediates the cellular response to curcumin. The observation that the effect of curcumin on cell proliferation and down-regulation of catalase and SOD were dependent on PKA further supports the idea that curcumin does not act directly to increase ROS in the cells. PKA is a major regulator during multicellular development in *D*. *discoideum* and all metazoans [63]. Although PKA is expressed in proliferating *D*. *discoideum* cells [77], PKA null cells divide normally [78, 79]. Thus, this demonstration that PKA functions to regulate the response of cells to a xenobiotic suggests that this may be a normal role for the enzyme in mitotically dividing cells.

**Fig 13:**
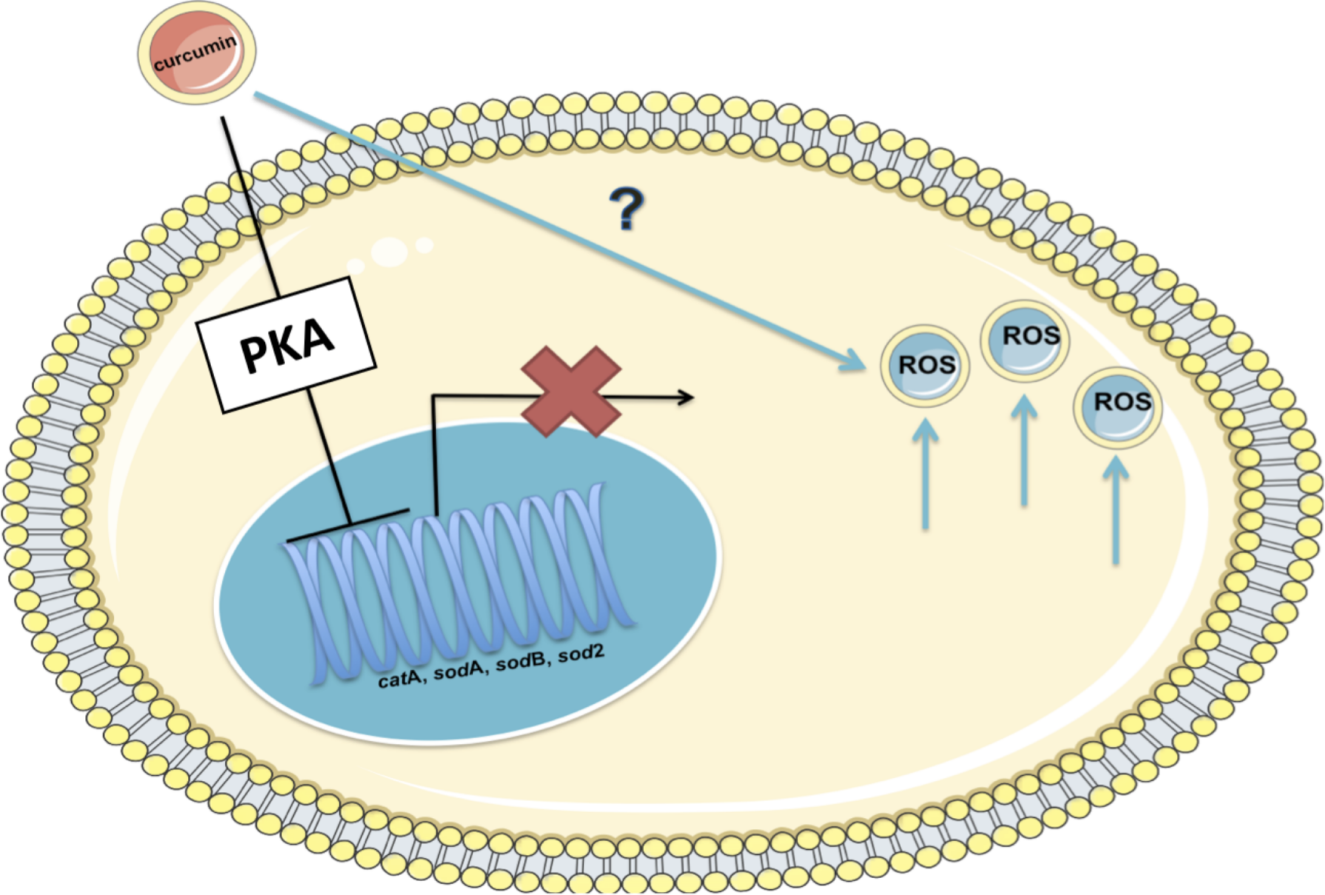
Proposed mechanism of curcumin action. Curcumin inhibits growth and generates ROS in *D*. *discoideum*. Curcumin induced major changes in transcription which included the reduction of catalase A and superoxide dismutase enzyme levels through a PKA mediated pathway. The results of this study suggest that the increase in ROS is not the cause of the decrease in antioxidant enzyme levels, but rather that the decrease in the enzymes results in the increase in ROS levels.

Studies in *D*. *discoideum* on the effects of valproic acid, a bipolar disorder treatment, have shown that the cellular response to the drugs is mediated though PKA [31]. This was subsequently translated and verified in mammalian cells. In addition, PKA has been shown to mediate sensitivity to the widely used anti-cancer drug cisplatin [66, 80]. Overall, the current studies reveal a novel mechanism by which curcumin also affects cell physiology (Fig 13).

These findings prompted the use of RNA-seq to provide an unbiased and more nuanced view of the transcriptional effects of curcumin. The results showed that there is an early and transient dose dependent effect on transcription that affects about 700 genes (approximately 5% of the genome). This early response to curcumin is limited compared to the transcriptional response accompanying the transition to development in *D*. *discoideum* where the transcription of about one-half of the genes is affected [70].

Curcumin quickly up-regulates the transcription of 533 genes that encode oxidoreductase enzymes, osmotic/salt/heat stress proteins, ABC transporters, and cell cycle proteins. The early down-regulated group includes genes involved with cell death, defense from bacteria, peroxisome function, and sphingomyelin catabolism. This early transcriptional response is transient and is replaced by a subsequent change in gene expression where 204 genes are up-regulated while 443 genes are down-regulated. The later up-regulated genes encode proteins involved in oxidoreductase activity, transcription factors, antioxidant activity, vitamin binding and the response to abiotic stimuli. The down-regulated late genes include those involved with the cells cycle, DNA replication, response to drugs, as well as oxidoreductase activity. The GO term enrichment lists at the early and late time points differ and further emphasize the cellular response to curcumin is multiphasic and complex.

This pleiotropy of the transcriptional response makes it challenging to construct a unifying hypothesis. However, some categories of genes suggest fruitful paths forward. Oxidoreductases are among the most prominent differentially expressed genes, and their broad spectrum of substrates makes them particularly interesting in the context of the cellular response to curcumin. The transcription factors represent an interesting target and detailed study may reveal conserved target genes for the factors leading to a clearer picture of how curcumin affects cells. ABC transporters are also of interest in that they are central to the response to many chemicals, and that there are *D*. *discoideum* mutants in the transporters available [81].

Genome wide analysis including microarrays, RNAseq, and proteomics have been used previously to examine the effect of curcumin alone, or in combination with other compounds, in a variety of cell and tissue types (see review [82]). Examples of RNAseq studies include the effect of curcumin on a mouse model of colorectal cancer [83], parasitic mites [84], and breast cancer stem cells [85]. Microarray studies characterized the effect of curcumin on lung [86, 87] and breast [88] cancer cell lines. Proteomic studies included studies on HCT116 colon cancer cells [89] and HepG2 cells [90].

Although these studies represent a broad range of biological interest, they all report a widely pleiotropic effect on transcription or protein expression. Moreover, the studies indicate that the transcripts affected by curcumin encode proteins involved in many different biological functions and pathways and are localized in many different cellular compartments. Some of these (cell death, oxidoreductase, cell cycle control) are of the same functional type as was found in *D*. *discoideum* suggesting some conservation of underlying mechanism. It is important to re-emphasize the genetic tractability of this system that provides a way to follow up on these observations.

Our studies extend a previous study focused on the effect of curcumin on multicellular development of *D*. *discoideum* [91]. The authors showed that curcumin inhibited both chemotaxis and multicellular development. The accumulation of the mRNAs of the early developmentally regulated genes *yakA*, *keaA*, *pkaC* and *carA* was also inhibited, while two of five glutathione-S-transferase genes were up-regulated. Our finding that cell proliferation of the *pkaC* null and the *pkaR*-over-expressing mutants is more resistant to the inhibitory effects of curcumin emphasizes the importance of this enzyme as a central regulator in the response to curcumin both in cell proliferation and development in this organism. Both studies show pleiotropic effects of curcumin, but there appear to be significant differences of the effects of curcumin on proliferating and developing cells.

Further work needs to address the precise mechanism by which curcumin affects PKA and how PKA in turn affects transcription. Indeed, the mechanism by which the groups of early and late genes are transcriptionally controlled remains to be answered. In addition, these studies suggest new functions for genes (e.g., oxidoreductases, ABC transporters, and transcription factors) that can be further investigated by making the appropriate null mutations.

The significant and unexpected complexity of the effect of curcumin on cell physiology revealed by these studies underscores the possibility of negative effects and unknown drug interactions when it is taken prophylactically or therapeutically. Indeed, there is no rational dose for the human consumption of curcumin as there is no known effect or measurable endpoint. The data suggest that the complex transcriptional effect of curcumin on cells may be a severely confounding effect in many previous studies [27].

Overall, these studies provide a new look at the effect of curcumin on cells and opens up new avenues for investigation. As in earlier drug studies employing *D*. *discoideum*, these ideas can be extended to human cells [31, 92, 93].

## Supporting information

S1 Fig Reproducibility of biological replicates in RNA-seq data. For the RNA-seq analysis, each treatment (time and concentration) was tested in triplicate. We evaluated the reproducibility between any two biological replicates by computing Spearman’s correlations. We plotted the correlation between each two of the three biological replicates in each treatment (as defined by concentration and time). Blue diamonds, red squares and green triangles represent the correlations between replicates 1 and 2, 1 and 3, and 2 and 3, respectively. Most of correlations between any two biological replicates at each time point are higher than 0.98, except for two samples: rep2 treated with 7.5 μg/ml at 8 hours and rep2 treated with 2.5 μg/ml at 12 hours. These samples exhibited lower correlations (around 0.95, blue diamond and green triangle) with the other two replicates. The correlation between any two samples of different concentration or different time was always higher than 0.95 (data not shown). Therefore, we removed the least-correlated samples (rep2 treated with 7.5 μg/ml at 8 hours and rep2 treated with 2.5 μg/ml at 12 hours) from further analysis.
(PDF)

S1 Table D. discoideum strains used in this study. ߡ = Null gene, OE = overexpressed gene, AX4/AX2 = WT.
(DOCX)

S2 Table Primers used for the qRT-PCR in this study. (PDF)

S3 Table Selected gene ontology (GO) enrichment data of differentially expressed genes in response to short exposure to curcumin. A) 533 genes were identified as up-regulated upon early exposure (4 hours) to high concentration (10 μg/ml) of curcumin. GO enrichment analysis revealed the genes are involved in various functions including oxidoreductase activity, response to osmotic/salt/heat stress and the cell cycle. Eleven ABC transporters are also included. B) 145 genes were identified as down-regulated upon early exposure (4 hours) to high concentration (10 μg/ml) of curcumin. GO analysis revealed the genes are involved in functions including hydrolase activity, sphingomyelin catabolism, apoptosis, defense response to bacterium, and peroxisome function.
(PDF)

S4 Table Selected GO enrichment data of differentially expressed genes upon extended exposure to curcumin. A) 204 genes up-regulated upon extended exposure (12 hours) to high concentration (10 μg/ml) of curcumin are involved in various functions including oxidoreductase activity, antioxidant activity, vitamin binding, response to abiotic stimulus, and contractile vacuole. Seven ABC transporters and 6 transcription factors including STATb and STATc, are also included. B) 443 genes down-regulated upon extended exposure to curcumin are involved in various functions including cell cycle control, DNA replication and responses to drugs. Eight genes that encode cytochrome P450 family proteins, which generally have a terminal oxidoreductase activity, are also included. Note that *carA-1*, *pkaR* and *regA*, which are involved in cAMP-mediated signaling, are also down-regulated upon extended exposure.
(PDF)

S5 Table Selected GO enrichment data of differentially expressed genes during growth without exposure to curcumin. **A)** 67 genes are up-regulated during growth without exposure to curcumin. The GO term enrichment analysis revealed mostly cell cycle related genes. B) 23 genes are down-regulated during growth without exposure to curcumin.
(PDF)

S6 Table Selected GO enrichment data of genes which are differentially expresssed during growth only under curcumin treatment. A) Genes up-regulated without curcumin (S5 Table) were omitted from the GO term enrichment analysis to focus on genes up-regulated due to curcumin exposure during growth. 161 genes were up-regulated at 12 hours only under curcumin treatment. Genes related to oxidoreductase activity, gultathione transferase activity, oxygen transport, response to oxidative stress, and sphingolipid catabolic process were enriched in the gene set. B) 733 genes were down-regulated under curcumin treatment. Genes related to DNA replication, oxidoreductase activity and contractile vacuolar membrane, are enriched in the gene set. Genes that encode cytochrome P450 family proteins, which generally have a terminal oxidoreductase activity, and genes involved in cAMP-mediated signaling, *carA-1*, *pkaR* and *regA*, are also down-regulated upon extended exposure.
(PDF)

S7 Table Selected GO enrichment data of up-regulated genes at all time points under curcumin treatment. Among the genes that are differentially expressed only under curcumin treatment (S6 Table), 50 genes were always expressed at higher levels (at least 2 fold) in the cells treated with high concentration (10 μg/ml) of curcumin. Genes involved in antioxidant activity were highly enriched. Note that most of the genes exhibited peak expression at an early time point (4 hours, refer to Fig 12E).
(PDF)

## References

[1] Herbal medicine in the United States: Review of efficacy, safety, and regulation—Grand Rounds at University of California, San Francisco Medical Center, (2008). 10.1007/s11606-008-0632-y PMC2517879 18415652

[2] Research, Markets. Research and Markets: Global Market Study on Botanical Supplements 2014–2020—Personal Care Segment to Witness Highest Growth in the $90 Billion Market. 2015.

[3] Beevers CH, S. Pharmacological and clinical properties of curcumin. Botanics: Targets and Therapy. 2011;(1):5–18.

[4] Jayaprakasha GK, Jaganmohan Rao L, Sakariah KK. Antioxidant activities of curcumin, demethoxycurcumin and bisdemethoxycurcumin. Food Chemistry. 2006;98:720–4. doi: 10.1016/j.foodchem.2005.06.037

[5] Sharma RA, Gescher AJ, Steward WP. Curcumin: the story so far. Eur J Cancer. 2005;41(13):1955–68. Epub 2005/08/06. doi: 10.1016/j.ejca.2005.05.009 . 16081279 10.1016/j.ejca.2005.05.009

[6] Conney AH, Lysz T, Ferraro T, Abidi TF, Manchand PS, Laskin JD, et al Inhibitory effect of curcumin and some related dietary compounds on tumor promotion and arachidonic acid metabolism in mouse skin. Adv Enzyme Regul. 1991;31:385–96. . 1908616 10.1016/0065-2571(91)90025-h

[7] Anand P, Sundaram C, Jhurani S, Kunnumakkara AB, Aggarwal BB. Curcumin and cancer: An "old-age" disease with an "age-old" solution. Cancer Letters. 2008;267:133–64. doi: 10.1016/j.canlet.2008.03.025 . 18462866 10.1016/j.canlet.2008.03.025

[8] Libby P. Inflammation in atherosclerosis. Nature. 2002;420:868–74. doi: 10.1038/nature01323 . 12490960 10.1038/nature01323

[9] Lumeng CN, Saltiel AR. Inflammatory links between obesity and metabolic disease. Journal of Clinical Investigation. 2011;121:2111–7. doi: 10.1172/JCI57132 . 21633179 10.1172/JCI57132 PMC3104776

[10] Coussens LM, Werb Z. Inflammation and cancer. Nature. 2002;420(6917):860–7. doi: 10.1038/nature01322 ; PubMed Central PMCID: PMCPMC2803035. 12490959 10.1038/nature01322 PMC2803035

[11] Balkwill F, Mantovani A. Inflammation and cancer: back to Virchow? Lancet. 2001;357(9255):539–45. doi: 10.1016/S0140-6736(00)04046-0 . 11229684 10.1016/S0140-6736(00)04046-0

[12] Singh S, Aggarwal BB. Activation of transcription factor NF-kappa B is suppressed by curcumin (diferuloylmethane) [corrected]. The Journal of biological chemistry. 1995;270:24995–5000. doi: 10.1074/jbc.270.50.30235 . 7559628 10.1074/jbc.270.42.24995

[13] Lal B, Kapoor AK, Asthana OP, Agrawal PK, Prasad R, Kumar P, et al Efficacy of curcumin in the management of chronic anterior uveitis. Phytotherapy Research. 1999;13:318–22. doi: 10.1002/(SICI)1099-1573(199906)13:4<318::AID-PTR445>3.0.CO;2-7 . 10404539 10.1002/(SICI)1099-1573(199906)13:4<318::AID-PTR445>3.0.CO;2-7

[14] Takada Y, Bhardwaj A, Potdar P, Aggarwal BB. Nonsteroidal anti-inflammatory agents differ in their ability to suppress NF-kappaB activation, inhibition of expression of cyclooxygenase-2 and cyclin D1, and abrogation of tumor cell proliferation. Oncogene. 2004;23:9247–58. doi: 10.1038/sj.onc.1208169 . 15489888 10.1038/sj.onc.1208169

[15] Mishra S, Palanivelu K. The effect of curcumin (turmeric) on Alzheimer's disease: An overview. Ann Indian Acad Neurol. 2008;11:13–9. doi: 10.4103/0972-2327.40220 . 19966973 10.4103/0972-2327.40220 PMC2781139

[16] Zhang L, Fiala M, Cashman J, Sayre J, Espinosa A, Mahanian M, et al Curcuminoids enhance amyloid-beta uptake by macrophages of Alzheimer's disease patients. Journal of Alzheimer's disease: JAD. 2006;10:1–7. . 16988474 10.3233/jad-2006-10101

[17] Harman D. Aging: a theory based on free radical and radiation chemistry. Journal of gerontology. 1956;11:298–300. doi: 10.1093/geronj/11.3.298 . 13332224 10.1093/geronj/11.3.298

[18] Halliwell B, Gutteridge JMC. Free Radicals in Biology and Medicine. Free Radical Biology and Medicine. 1999;10:449–50. doi: 10.1016/0891-5849(91)90055-8 PubMed PMID: 6297065.

[19] McCord JM, Keele BB, Fridovich I. An enzyme-based theory of obligate anaerobiosis: the physiological function of superoxide dismutase. Proceedings of the National Academy of Sciences of the United States of America. 1971;68:1024–7. doi: 10.1073/pnas.68.5.1024 . 4995818 10.1073/pnas.68.5.1024 PMC389105

[20] Herrera E, Barbas C. Vitamin E: action, metabolism and perspectives. Journal of physiology and biochemistry. 2001;57:43–56. doi: 10.1007/BF03179812 . 11579997

[21] Ames BN, Cathcart R, Schwiers E, Hochstein P. Uric acid provides an antioxidant defense in humans against oxidant- and radical-caused aging and cancer: a hypothesis. Proceedings of the National Academy of Sciences of the United States of America. 1981;78:6858–62. doi: 10.1073/pnas.78.11.6858 . 6947260 10.1073/pnas.78.11.6858 PMC349151

[22] Joe B, Lokesh BR. Role of capsaicin, curcumin and dietary n-3 fatty acids in lowering the generation of reactive oxygen species in rat peritoneal macrophages. Biochimica et biophysica acta. 1994;1224:255–63. 0167-4889(94)90198-8 [pii]. . 7981240 10.1016/0167-4889(94)90198-8

[23] Kunchandy ER MN. Oxygen radical scavenging activity of curcumin. International Journal of Pharmaceutics. 1990;58(3):237–40.

[24] Tcnnesen HH, Greenhill JV. Studies on curcumin and curcuminoids. XXII: Curcumin as a reducing agent and as a radical scavenger. International Journal of Pharmaceutics. 1992;87:79–87. doi: 10.1016/0378-5173(92)90230-Y

[25] Khan MA, Gahlot S. Oxidative Stress Induced by Curcumin Promotes the Death of Cutaneous T-cell Lymphoma (HuT-78) by Disrupting the Function of Several Molecular Targets. Mol Cancer Ther. 2012;11:1873–83. doi: 10.1158/1535-7163.MCT-12-0141 22653966 10.1158/1535-7163.MCT-12-0141

[26] Yoshino M, Haneda M, Naruse M, Htay HH, Tsubouchi R, Qiao SL, et al Prooxidant activity of curcumin: Copper-dependent formation of 8-hydroxy-2′-deoxyguanosine in DNA and induction of apoptotic cell death. Toxicology in Vitro. 2004;18:783–9. doi: 10.1016/j.tiv.2004.03.009 . 15465643 10.1016/j.tiv.2004.03.009

[27] Nelson KM, Dahlin JL, Bisson J, Graham J, Pauli GF, Walters MA. The Essential Medicinal Chemistry of Curcumin. J Med Chem. 2017 doi: 10.1021/acs.jmedchem.6b00975 . 28074653 10.1021/acs.jmedchem.6b00975 PMC5346970

[28] Misty R, Martinez R, Ali H, Steimle PA. Naringenin is a novel inhibitor of Dictyostelium cell proliferation and cell migration. Biochem Biophys Res Commun. 2006;345(1):516–22. doi: 10.1016/j.bbrc.2006.04.047 . 16682000 10.1016/j.bbrc.2006.04.047

[29] Waheed A, Ludtmann MH, Pakes N, Robery S, Kuspa A, Dinh C, et al Naringenin inhibits the growth of Dictyostelium and MDCK-derived cysts in a TRPP2 (polycystin-2)-dependent manner. Br J Pharmacol. 2014;171(10):2659–70. doi: 10.1111/bph.12443 ; PubMed Central PMCID: PMCPMC4009007. 24116661 10.1111/bph.12443 PMC4009007

[30] Alexander S, Min J, Alexander H. *Dictyostelium discoideum* to human cells: pharmacogenetic studies demonstrate a role for sphingolipids in chemoresistance. Biochimica et biophysica acta. 2006;1760:301–9. doi: 10.1016/j.bbagen.2005.11.015 . 16403600 10.1016/j.bbagen.2005.11.015

[31] Boeckeler K, Adley K, Xu X, Jenkins A, Jin T, Williams RS. The neuroprotective agent, valproic acid, regulates the mitogen-activated protein kinase pathway through modulation of protein kinase A signalling in *Dictyostelium discoideum*. Eur J Cell Biol. 2006;85(9–10):1047–57. doi: 10.1016/j.ejcb.2006.04.013 . 16759735 10.1016/j.ejcb.2006.04.013

[32] Kessin RH. *Dictyostelium*: evolution, cell biology, and the development of multicellularity Cambridge, UK; New York: Cambridge University Press; 2001 xiv, 294 p. p.

[33] Alexander S, Alexander H. Lead genetic studies in *Dictyostelium discoideum* and translational studies in human cells demonstrate that sphingolipids are key regulators of sensitivity to cisplatin and other anticancer drugs. Semin Cell Dev Biol. 2011;22(1):97–104. doi: 10.1016/j.semcdb.2010.10.005 . 20951822 10.1016/j.semcdb.2010.10.005

[34] Ludtmann MH, Boeckeler K, Williams RS. Molecular pharmacology in a simple model system: implicating MAP kinase and phosphoinositide signalling in bipolar disorder. Semin Cell Dev Biol. 2011;22(1):105–13. doi: 10.1016/j.semcdb.2010.11.002 ; PubMed Central PMCID: PMCPMC3032892. 21093602 10.1016/j.semcdb.2010.11.002 PMC3032892

[35] Sussman M. Cultivation and synchronous morphogenesis of *Dictyostelium* under controlled experimental conditions. Methods Cell Biol. 1987;28:9–29. . 3298997 10.1016/s0091-679x(08)61635-0

[36] Min J, Sridevi P, Alexander S, Alexander H. Sensitive cell viability assay for use in drug screens and for studying the mechanism of action of drugs in *Dictyostelium discoideum*. Biotechniques. 2006;41(5):591–5. Epub 2006/12/05. . 17140116 10.2144/000112260

[37] Garcia MX, Foote C, van Es S, Devreotes PN, Alexander S, Alexander H. Differential developmental expression and cell type specificity of *Dictyostelium* catalases and their response to oxidative stress and UV-light. Biochimica et biophysica acta. 2000;1492:295–310. doi: 10.1016/S0167-4781(00)00063-4 . 11004503 10.1016/s0167-4781(00)00063-4

[38] Martin JP Jr., Dailey M, Sugarman E. Negative and positive assays of superoxide dismutase based on hematoxylin autoxidation. Arch Biochem Biophys. 1987;255(2):329–36. . 3036004 10.1016/0003-9861(87)90400-0

[39] Able AJ, Guest DI, Sutherland MW. Use of a new tetrazolium-based assay to study the production of superoxide radicals by tobacco cell cultures challenged with avirulent zoospores of phytophthora parasitica var nicotianae. Plant Physiol. 1998;117(2):491–9. ; PubMed Central PMCID: PMCPMC34969. 9625702 10.1104/pp.117.2.491 PMC34969

[40] Ukeda H, Maeda S, Ishii T, Sawamura M. Spectrophotometric assay for superoxide dismutase based on tetrazolium salt 3'—1—(phenylamino)-carbonyl—3, 4-tetrazolium]-bis(4-methoxy-6-nitro)benzenesulfonic acid hydrate reduction by xanthine-xanthine oxidase. Anal Biochem. 1997;251(2):206–9. doi: 10.1006/abio.1997.2273 . 9299017 10.1006/abio.1997.2273

[41] Bloomfield G, Pears C. Superoxide signalling required for multicellular development of *Dictyostelium*. Journal of cell science. 2003;116(Pt 16):3387–97. doi: 10.1242/jcs.00649 . 12840076 10.1242/jcs.00649

[42] Teo R, Lewis KJ, Forde JE, Ryves WJ, Reddy JV, Rogers BJ, et al Glycogen synthase kinase-3 is required for efficient *Dictyostelium* chemotaxis. Mol Biol Cell. 2010;21(15):2788–96. doi: 10.1091/mbc.E09-10-0891 ; PubMed Central PMCID: PMCPMC2912363. 20534815 10.1091/mbc.E09-10-0891 PMC2912363

[43] Huang E, Talukder S, Hughes TR, Curk T, Zupan B, Shaulsky G, et al BzpF is a CREB-like transcription factor that regulates spore maturation and stability in Dictyostelium. Dev Biol. 2011;358(1):137–46. doi: 10.1016/j.ydbio.2011.07.017 ; PubMed Central PMCID: PMCPMC3180911. 21810415 10.1016/j.ydbio.2011.07.017 PMC3180911

[44] Miranda ER, Rot G, Toplak M, Santhanam B, Curk T, Shaulsky G, et al Transcriptional profiling of *Dictyostelium* with RNA sequencing. Methods Mol Biol. 2013;983:139–71. doi: 10.1007/978-1-62703-302-2_8 ; PubMed Central PMCID: PMCPMC3892559. 23494306 10.1007/978-1-62703-302-2_8 PMC3892559

[45] Santhanam B, Cai H, Devreotes PN, Shaulsky G, Katoh-Kurasawa M. The GATA transcription factor GtaC regulates early developmental gene expression dynamics in Dictyostelium. Nat Commun. 2015;6:7551 doi: 10.1038/ncomms8551 ; PubMed Central PMCID: PMCPMC4506546. 26144553 10.1038/ncomms8551 PMC4506546

[46] Katoh-Kurasawa M, Santhanam B, Shaulsky G. The GATA transcription factor gene gtaG is required for terminal differentiation in Dictyostelium. Journal of cell science. 2016 doi: 10.1242/jcs.181545 ; PubMed Central PMCID: PMCPMC4852770. 26962009 10.1242/jcs.181545 PMC4852770

[47] Tonnesen HH, Karlsen J. Studies on curcumin and curcuminoids. VI. Kinetics of curcumin degradation in aqueous solution. Z Lebensm Unters Forsch. 1985;180(5):402–4. Epub 1985/05/01. . 4013525 10.1007/BF01027775

[48] Kunchandy E, Rao MN. Oxygen radical scavenging activity of curcumin. International Journal of Pharmaceutics. 1990; 58:237–240.

[49] Mates JM. Effects of antioxidant enzymes in the molecular control of reactive oxygen species toxicology. Toxicology. 2000;153(1–3):83–104. . 11090949 10.1016/s0300-483x(00)00306-1

[50] Weydert CJ, Cullen JJ. Measurement of superoxide dismutase, catalase and glutathione peroxidase in cultured cells and tissue. Nat Protoc. 2010;5(1):51–66. doi: 10.1038/nprot.2009.197 ; PubMed Central PMCID: PMCPMC2830880. 20057381 10.1038/nprot.2009.197 PMC2830880

[51] Garcia MX, Alexander H, Mahadeo D, Cotter DA, Alexander S. The *Dictyostelium discoideum* prespore-specific catalase B functions to control late development and to protect spore viability. Biochim Biophys Acta. 2003;1641(1):55–64. Epub 2003/06/06. . 12788229 10.1016/s0167-4889(03)00064-8

[52] Aggeli IK, Koustas E, Gaitanaki C, Beis I. Curcumin acts as a pro-oxidant inducing apoptosis via JNKs in the isolated perfused Rana ridibunda heart. J Exp Zool A Ecol Genet Physiol. 2013;319(6):328–39. doi: 10.1002/jez.1797 . 23630153 10.1002/jez.1797

[53] Mates JM, Perez-Gomez C, Nunez de Castro I. Antioxidant enzymes and human diseases. Clin Biochem. 1999;32(8):595–603. . 10638941 10.1016/s0009-9120(99)00075-2

[54] Murata H, Takamatsu H, Liu S, Kataoka K, Huh NH, Sakaguchi M. NRF2 Regulates PINK1 Expression under Oxidative Stress Conditions. PLoS One. 2015;10(11):e0142438 doi: 10.1371/journal.pone.0142438 ; PubMed Central PMCID: PMCPMC4640816. 26555609 10.1371/journal.pone.0142438 PMC4640816

[55] Garcia MX, Roberts C, Alexander H, Stewart AM, Harwood A, Alexander S, et al Methanol and acriflavine resistance in *Dictyostelium* are caused by loss of catalase. Microbiology. 2002;148(Pt 1):333–40. Epub 2002/01/10. doi: 10.1099/00221287-148-1-333 . 11782526 10.1099/00221287-148-1-333

[56] Souza GM, Lu S, Kuspa A. YakA, a protein kinase required for the transition from growth to development in *Dictyostelium*. Development. 1998;125(12):2291–302. Epub 1998/05/19. . 9584128 10.1242/dev.125.12.2291

[57] Taminato A, Bagattini R, Gorjao R, Chen G, Kuspa A, Souza GM. Role for YakA, cAMP, and protein kinase A in regulation of stress responses of *Dictyostelium discoideum* cells. Mol Biol Cell. 2002;13(7):2266–75. Epub 2002/07/23. doi: 10.1091/mbc.01-11-0555 ; PubMed Central PMCID: PMC117311. 12134067 10.1091/mbc.01-11-0555 PMC117311

[58] Moussavi M, Assi K, Gomez-Munoz A, Salh B. Curcumin mediates ceramide generation via the de novo pathway in colon cancer cells. Carcinogenesis. 2006;27(8):1636–44. doi: 10.1093/carcin/bgi371 . 16501251 10.1093/carcin/bgi371

[59] Shakor AB, Atia M, Ismail IA, Alshehri A, El-Refaey H, Kwiatkowska K, et al Curcumin induces apoptosis of multidrug-resistant human leukemia HL60 cells by complex pathways leading to ceramide accumulation. Biochim Biophys Acta. 2014;1841(12):1672–82. doi: 10.1016/j.bbalip.2014.09.006 . 25240837 10.1016/j.bbalip.2014.09.006

[60] Yang YL, Ji C, Cheng L, He L, Lu CC, Wang R, et al Sphingosine kinase-1 inhibition sensitizes curcumin-induced growth inhibition and apoptosis in ovarian cancer cells. Cancer Sci. 2012;103(8):1538–45. doi: 10.1111/j.1349-7006.2012.02335.x . 22594559 10.1111/j.1349-7006.2012.02335.x PMC7659178

[61] Min J, Traynor D, Stegner AL, Zhang L, Hanigan MH, Alexander H, et al Sphingosine kinase regulates the sensitivity of *Dictyostelium discoideum* cells to the anticancer drug cisplatin. Eukaryot Cell. 2005;4(1):178–89. Epub 2005/01/12. doi: 10.1128/EC.4.1.178-189.2005 ; PubMed Central PMCID: PMC544159. 15643073 10.1128/EC.4.1.178-189.2005 PMC544159

[62] Segall JE, Kuspa A, Shaulsky G, Ecke M, Maeda M, Gaskins C, et al A MAP kinase necessary for receptor-mediated activation of adenylyl cyclase in *Dictyostelium*. J Cell Biol. 1995;128(3):405–13. ; PubMed Central PMCID: PMCPMC2120359. 7844154 10.1083/jcb.128.3.405 PMC2120359

[63] Loomis WF. Role of PKA in the timing of developmental events in *Dictyostelium* cells. Microbiol Mol Biol Rev. 1998;62(3):684–94. ; PubMed Central PMCID: PMCPMC98931. 9729606 10.1128/mmbr.62.3.684-694.1998 PMC98931

[64] Shaulsky G, Escalante R, Loomis WF. Developmental signal transduction pathways uncovered by genetic suppressors. Proc Natl Acad Sci U S A. 1996;93(26):15260–5. ; PubMed Central PMCID: PMCPMC26391. 8986798 10.1073/pnas.93.26.15260 PMC26391

[65] Shaulsky G, Fuller D, Loomis WF. A cAMP-phosphodiesterase controls PKA-dependent differentiation. Development. 1998;125(4):691–9. . 9435289 10.1242/dev.125.4.691

[66] Li G, Alexander H, Schneider N, Alexander S. Molecular basis for resistance to the anticancer drug cisplatin in *Dictyostelium*. Microbiology. 2000;146 (Pt 9):2219–27. Epub 2000/09/07. doi: 10.1099/00221287-146-9-2219 . 10974109 10.1099/00221287-146-9-2219

[67] Mann SK, Brown JM, Briscoe C, Parent C, Pitt G, Devreotes PN, et al Role of cAMP-dependent protein kinase in controlling aggregation and postaggregative development in *Dictyostelium*. Dev Biol. 1997;183(2):208–21. doi: 10.1006/dbio.1996.8499 . 9126295 10.1006/dbio.1996.8499

[68] Mutzel R, Lacombe ML, Simon MN, de Gunzburg J, Veron M. Cloning and cDNA sequence of the regulatory subunit of cAMP-dependent protein kinase from *Dictyostelium discoideum*. Proc Natl Acad Sci U S A. 1987;84(1):6–10. ; PubMed Central PMCID: PMCPMC304130. 3467359 10.1073/pnas.84.1.6 PMC304130

[69] Harwood AJ, Hopper NA, Simon MN, Bouzid S, Veron M, Williams JG. Multiple roles for cAMP-dependent protein kinase during *Dictyostelium* development. Dev Biol. 1992;149(1):90–9. . 1728597 10.1016/0012-1606(92)90266-j

[70] Parikh A, Miranda ER, Katoh-Kurasawa M, Fuller D, Rot G, Zagar L, et al Conserved developmental transcriptomes in evolutionarily divergent species. Genome Biol. 2010;11(3):R35 10.1186/gb-2010-11-3-r35. 20236529; PubMed Central PMCID: PMCPMC2864575. doi: 10.1186/gb-2010-11-3-r35 20236529 10.1186/gb-2010-11-3-r35 PMC2864575

[71] Van Driessche N, Demsar J, Booth EO, Hill P, Juvan P, Zupan B, et al Epistasis analysis with global transcriptional phenotypes. Nat Genet. 2005;37(5):471–7. 10.1038/ng1545. 15821735. doi: 10.1038/ng1545 15821735 10.1038/ng1545

[72] Hatcher H, Planalp R, Cho J, Torti FM, Torti SV. Curcumin: from ancient medicine to current clinical trials. Cell Mol Life Sci. 2008;65(11):1631–52. doi: 10.1007/s00018-008-7452-4 ; PubMed Central PMCID: PMCPMC4686230. 18324353 10.1007/s00018-008-7452-4 PMC4686230

[73] Williams RSB. Pharmacogenetics in model systems: defining a common mechanism of action for mood stabilisers. Progress in neuro-psychopharmacology & biological psychiatry. 2005;29:1029–37. doi: 10.1016/j.pnpbp.2005.03.020 . 15950352 10.1016/j.pnpbp.2005.03.020 PMC1249490

[74] Deng HX, Hentati A, Tainer JA, Iqbal Z, Cayabyab A, Hung WY, et al Amyotrophic lateral sclerosis and structural defects in Cu,Zn superoxide dismutase. Science. 1993;261(5124):1047–51. . 8351519 10.1126/science.8351519

[75] Kuspa A, Loomis WF. Tagging developmental genes in *Dictyostelium* by restriction enzyme-mediated integration of plasmid DNA. Proc Natl Acad Sci U S A. 1992;89(18):8803–7. ; PubMed Central PMCID: PMCPMC50009. 1326764 10.1073/pnas.89.18.8803 PMC50009

[76] Li CL, Santhanam B, Webb AN, Zupan B, Shaulsky G. Gene discovery by chemical mutagenesis and whole-genome sequencing in Dictyostelium. Genome Res. 2016;26(9):1268–76. doi: 10.1101/gr.205682.116 ; PubMed Central PMCID: PMCPMC5052037. 27307293 10.1101/gr.205682.116 PMC5052037

[77] Leichtling BH, Majerfeld IH, Spitz E, Schaller KL, Woffendin C, Kakinuma S, et al A cytosolic cyclic AMP-dependent protein kinase in *Dictyostelium discoideum*. II. Developmental regulation. The Journal of biological chemistry. 1984;259(1):662–8. . 6323417

[78] Mann SK, Firtel RA. A developmentally regulated, putative serine/threonine protein kinase is essential for development in *Dictyostelium*. Mech Dev. 1991;35(2):89–101. . 1836954 10.1016/0925-4773(91)90060-j

[79] Simon MN, Pelegrini O, Veron M, Kay RR. Mutation of protein kinase A causes heterochronic development of *Dictyostelium*. Nature. 1992;356(6365):171–2. doi: 10.1038/356171a0 . 1312226 10.1038/356171a0

[80] Li G. Genes and pathways mediating the cytotoxicity of the anticancer drug cisplatin in *Dictyostelium discoideum*: University of Missouri; 2000.

[81] Miranda ER, Zhuchenko O, Toplak M, Santhanam B, Zupan B, Kuspa A, et al ABC transporters in Dictyostelium discoideum development. PLoS One. 2013;8(8):e70040 doi: 10.1371/journal.pone.0070040 ; PubMed Central PMCID: PMCPMC3743828. 23967067 10.1371/journal.pone.0070040 PMC3743828

[82] Huminiecki L, Horbanczuk J, Atanasov AG. The functional genomic studies of curcumin. Semin Cancer Biol. 2017 doi: 10.1016/j.semcancer.2017.04.002 . 28392463 10.1016/j.semcancer.2017.04.002

[83] Guo Y, Su ZY, Zhang C, Gaspar JM, Wang R, Hart RP, et al Mechanisms of colitis-accelerated colon carcinogenesis and its prevention with the combination of aspirin and curcumin: Transcriptomic analysis using RNA-seq. Biochem Pharmacol. 2017;135:22–34. doi: 10.1016/j.bcp.2017.02.021 ; PubMed Central PMCID: PMCPMC5541256. 28267439 10.1016/j.bcp.2017.02.021 PMC5541256

[84] Liu X, Wu D, Zhang Y, Zhou H, Lai T, Ding W. RNA-Seq Analysis Reveals Candidate Targets for Curcumin against Tetranychus cinnabarinus. Biomed Res Int. 2016;2016:2796260 doi: 10.1155/2016/2796260 ; PubMed Central PMCID: PMCPMC5031819. 27672652 10.1155/2016/2796260 PMC5031819

[85] Colacino JA, McDermott SP, Sartor MA, Wicha MS, Rozek LS. Transcriptomic profiling of curcumin-treated human breast stem cells identifies a role for stearoyl-coa desaturase in breast cancer prevention. Breast Cancer Res Treat. 2016;158(1):29–41. doi: 10.1007/s10549-016-3854-4 . 27306423 10.1007/s10549-016-3854-4 PMC5831404

[86] Chiang IT, Wang WS, Liu HC, Yang ST, Tang NY, Chung JG. Curcumin alters gene expression-associated DNA damage, cell cycle, cell survival and cell migration and invasion in NCI-H460 human lung cancer cells in vitro. Oncol Rep. 2015;34(4):1853–74. doi: 10.3892/or.2015.4159 . 26238775 10.3892/or.2015.4159

[87] Sahoo K, Dozmorov MG, Anant S, Awasthi V. The curcuminoid CLEFMA selectively induces cell death in H441 lung adenocarcinoma cells via oxidative stress. Invest New Drugs. 2012;30(2):558–67. doi: 10.1007/s10637-010-9610-4 ; PubMed Central PMCID: PMCPMC3110543. 21181232 10.1007/s10637-010-9610-4 PMC3110543

[88] Cine N, Limtrakul P, Sunnetci D, Nagy B, Savli H. Effects of curcumin on global gene expression profiles in the highly invasive human breast carcinoma cell line MDA-MB 231: A gene network-based microarray analysis. Exp Ther Med. 2013;5(1):23–7. doi: 10.3892/etm.2012.754 ; PubMed Central PMCID: PMCPMC3524226. 23251236 10.3892/etm.2012.754 PMC3524226

[89] Wang J, Zhang J, Zhang CJ, Wong YK, Lim TK, Hua ZC, et al In situ Proteomic Profiling of Curcumin Targets in HCT116 Colon Cancer Cell Line. Sci Rep. 2016;6:22146 doi: 10.1038/srep22146 ; PubMed Central PMCID: PMCPMC4768257. 26915414 10.1038/srep22146 PMC4768257

[90] Chen J, Li L, Su J, Li B, Zhang X, Chen T. Proteomic Analysis of G2/M Arrest Triggered by Natural Borneol/Curcumin in HepG2 Cells, the Importance of the Reactive Oxygen Species-p53 Pathway. J Agric Food Chem. 2015;63(28):6440–9. doi: 10.1021/acs.jafc.5b01773 . 26051007 10.1021/acs.jafc.5b01773

[91] Garige M, Walters E. Curcumin inhibits development and cell adhesion in *Dictyostelium discoideum*: Implications for YakA signaling and GST enzyme function. Biochem Biophys Res Commun. 2015;467(2):275–81. doi: 10.1016/j.bbrc.2015.09.175 . 26449461 10.1016/j.bbrc.2015.09.175

[92] Min J, Mesika A, Sivaguru M, Van Veldhoven PP, Alexander H, Futerman AH, et al (Dihydro)ceramide synthase 1 regulated sensitivity to cisplatin is associated with the activation of p38 mitogen-activated protein kinase and is abrogated by sphingosine kinase 1. Mol Cancer Res. 2007;5(8):801–12. Epub 2007/08/19. doi: 10.1158/1541-7786.MCR-07-0100 . 17699106 10.1158/1541-7786.MCR-07-0100

[93] Min J, Van Veldhoven PP, Zhang L, Hanigan MH, Alexander H, Alexander S. Sphingosine-1-phosphate lyase regulates sensitivity of human cells to select chemotherapy drugs in a p38-dependent manner. Mol Cancer Res. 2005;3(5):287–96. Epub 2005/05/12. doi: 10.1158/1541-7786.MCR-04-0197 . 15886300 10.1158/1541-7786.MCR-04-0197

