# Supplementary material for: Curcumin affects gene expression and reactive oxygen species via a PKA dependent mechanism in Dictyostelium discoideum
Source: PLoS One. 2017 Nov 14;12(11):e0187562. doi: 10.1371/journal.pone.0187562 (PMC5685611; doi:10.1371/journal.pone.0187562)
Supplement: S1 Fig — For the RNA-seq analysis, each treatment (time and concentration) was tested in triplicate. We evaluated the reproducibility between any two biological replicates by computing Spearman’s correlations. We plotted the correlation between each two of the three biological replicates in each treatment (as defined by concentration and time). Blue diamonds, red squares and green triangles represent the correlations between replicates 1 and 2, 1 and 3, and 2 and 3, respectively. Most of correlations between any two biological replicates at each time point are higher than 0.98, except for two samples: rep2 treated with 7.5 μg/ml at 8 hours and rep2 treated with 2.5 μg/ml at 12 hours. These samples exhibited lower correlations (around 0.95, blue diamond and green triangle) with the other two replicates. The correlation between any two samples of different concentration or different time was always higher than 0.95 (data not shown). Therefore, we removed the least-correlated samples (rep2 treated with 7.5 μg/ml at 8 hours and rep2 treated with 2.5 μg/ml at 12 hours) from further analysis. (PDF) [file pone.0187562.s001.pdf]

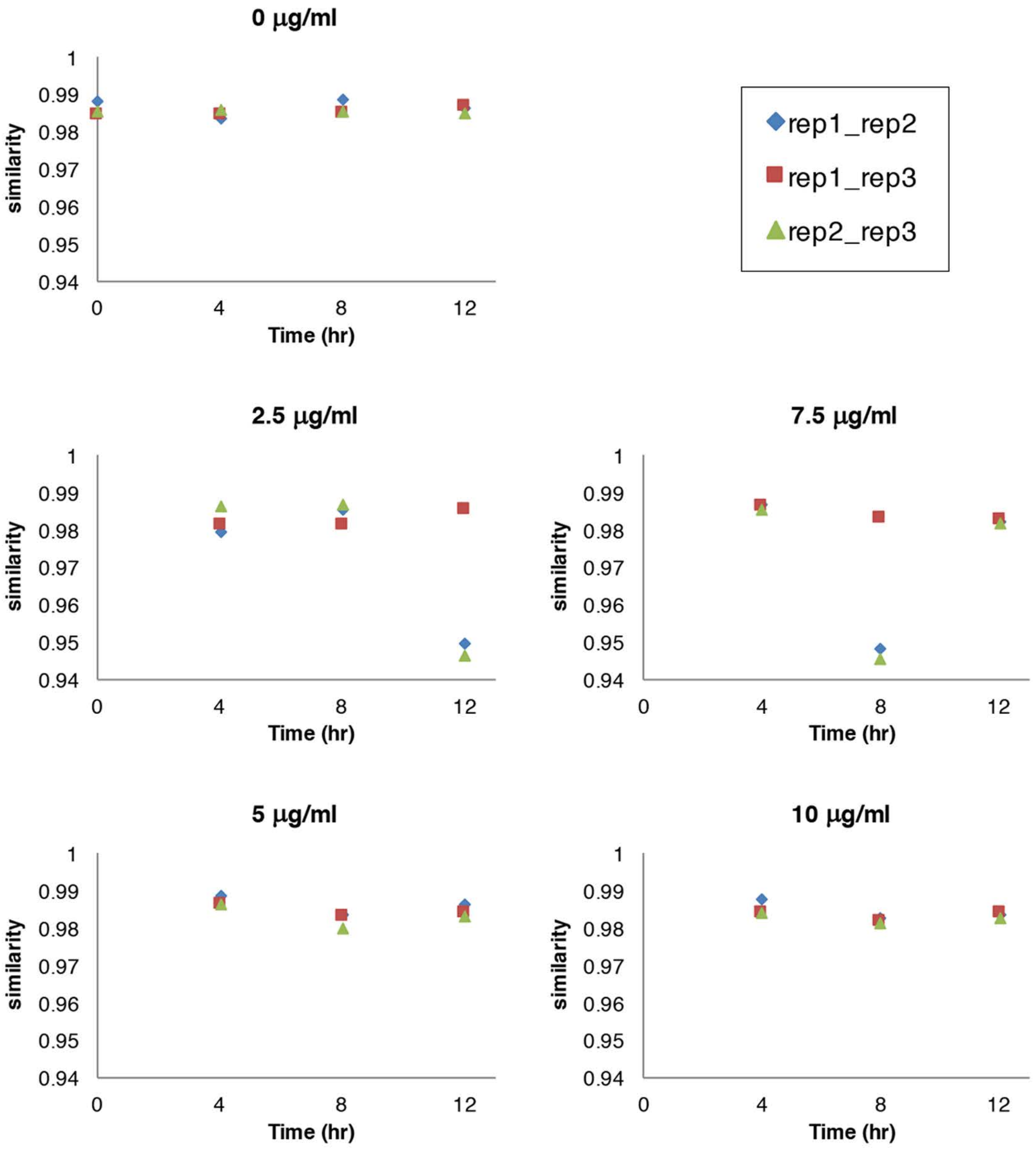

S1 Figure: Reproducibility of biological replicates in RNA-seq data.

For the RNA-seq analysis, each treatment (time and concentration) was tested in triplicates. We evaluated the reproducibility between any two biological replicates by computing Spearman's correlations. We plotted the correlation between each two of the three biological replicates in each treatment (as defined by concentration and time). Blue diamonds, red squares and green triangles represent the correlations between replicates 1 and 2, 1 and 3, and 2 and 3, respectively. Most of correlations between any two biological replicates at each time point are higher than 0.98, except for two samples: rep2 treated with 7.5 μg/ml at 8 hours and rep2 treated with 2.5 μg/ml at 12 hours. These samples exhibited lower correlations (around 0.95, blue diamond and green triangle) with the other two replicates. The correlation between any two samples of different concentration or different time was always higher than 0.95 (data not shown). Therefore, we removed the least-correlated samples (rep2 treated with 7.5 μg/ml at 8 hours and rep2 treated with 2.5 μg/ml at 12 hours) from further analysis.
