# Supplementary material for: Curcumin affects gene expression and reactive oxygen species via a PKA dependent mechanism in Dictyostelium discoideum
Source: PLoS One. 2017 Nov 14;12(11):e0187562. doi: 10.1371/journal.pone.0187562 (PMC5685611; doi:10.1371/journal.pone.0187562)
Supplement: S1 Table — ߡ = Null gene, OE = overexpressed gene, AX4/AX2 = WT. (DOCX) [file pone.0187562.s002.docx]

| NAME | GENOTYPE | PHENOTYPE | MUTAGENESIS METHOD | PARENT |
| --- | --- | --- | --- | --- |
| DBS0236257  (HM332) | *re*g*A*∆ | Aberrant chemotaxis to cAMP, aberrant fruiting body morphology, cisplatin resistant | Homologous recombination | AX4 |
| DBS0235464  (AK240) | *erkB*∆ | Abolished aggregation | Homologous recombination | AX4 |
| DBS0237193  (SA604) | *sgkA* OE | Aberrant cytokinesis, Increased resistance to cisplatin and carboplatin | Extrachromosomal | AX4 |
| DBS0266778 (IR41) | *catA*∆ | Abolished catalase A activity | Homologous recombination | AX4 |
| DBS0237087 | *yakA*∆ | Abolished aggregation | Homologous recombination | AX4 |
| DBS0237194 | *sodA* OE | Abolished aggregation, overexpressed SOD | Random insertion | AX2 |
| DBS0235411 | *pkaR*∆ | Abolished aggregation | Homologous Recombination | AX4 |
| DBS0236783 | *pkaC*∆ | Abolished aggregation, abolished PKA catalytic activity | Homologous recombination | JH10 (AX4) |
| DBS0235418 (4M) | *pkaR* OE | Neomycin resistant, PKA regulatory subunit overexpressor, axenic, abolished aggregation | Random insertion | AX2 |

**S1 Table. *D. discoideum* strains used in this study.** Δ = null genes, OE = overexpressed genes. AX4/AX2 = WT. DB numbers are the accession numbers in Dictybase.org.
