# Supplementary material for: Curcumin affects gene expression and reactive oxygen species via a PKA dependent mechanism in Dictyostelium discoideum
Source: PLoS One. 2017 Nov 14;12(11):e0187562. doi: 10.1371/journal.pone.0187562 (PMC5685611; doi:10.1371/journal.pone.0187562)
Supplement: S2 Table — (PDF) [file pone.0187562.s003.pdf]

Gene	Forward primer	Reverse Primer	Size
**IG7**	5'-CTC GAT GTC GGC TTA ACA CA-3'	5'-AGG GAC CAA ACT GTC TCA CG-3'	109
***cat*A**	5'-GCT GCT CGT CAA CCA TAC AA-3'	5'-CGG ACA TGT GAC CGA CTA AA -3'	121
***sod*A**	5'-GGT GAA CAC ACA ATC GTT GG-3'	5'-CCA ATG ACA CCA CAA CCA AG -3'	249
***sod*B**	5'-AAC CAC TGG CAA TGC AAA TA-3'	5'-CAA TAG CGG CAG ATG GAG AT -3'	110
***sod*E**	5'-TGG GTG TTT ATC AGC AGG AG-3'	5'-CCT AAA TCA CCA ACA TGA CGA -3'	90
***sod*2**	5'-GGT CCA TTG GCT GAT GCT AT-3'	5'-TAA CCA ACC CCA ACC AGA AC -3'	182

S2 Table: Primers used for qRT-PCR in this study.
