# Supplementary material for: Curcumin affects gene expression and reactive oxygen species via a PKA dependent mechanism in Dictyostelium discoideum
Source: PLoS One. 2017 Nov 14;12(11):e0187562. doi: 10.1371/journal.pone.0187562 (PMC5685611; doi:10.1371/journal.pone.0187562)
Supplement: S3 Table — A) 533 genes were identified as up-regulated upon early exposure (4 hours) to high concentration (10 μg/ml) of curcumin. GO enrichment analysis revealed the genes are involved in various functions including oxidoreductase activity, response to osmotic/salt/heat stress and the cell cycle. Eleven ABC transporters are also included. B) 145 genes were identified as down-regulated upon early exposure (4 hours) to high concentration (10 μg/ml) of curcumin. GO analysis revealed the genes are involved in functions including hydrolase activity, sphingomyelin catabolism, apoptosis, defense response to bacterium, and peroxisome function. (PDF) [file pone.0187562.s004.pdf]

A)

GO.ID	Term	Annotated	Significant	Expected	classic	fold enrichment	categ	genes
GO:0016491	oxidoreductase activity	513	50	24.39	5.90E-07	2.1	MF	aifB,alrE,chdh,cxgS,cyp508A4,cyp515B1,cyp516B1,iliB,malA,maoB-1, msrA,noxB,rnrA, 37 DDB_G genes
GO:0042626	ATPase activity, coupled to transmembran...	92	14	4.37	9.90E-05	3.2	MF	abcA2,abcB1,abcC12,abcC3,abcG11,abcG12,abcG14,abcG15,abcG17-1 abcG21,abcG9,DDB_G0267924,DDB_G0273235,DDB_G0289473
GO:0042803	protein homodimerization activity	29	6	1.38	0.00207	4.3	MF	abpC,csaA,dimB,dstC,mhcA,pefA
GO:0003825	alpha,alpha-trehalose-phosphate synthase...	2	2	0.1	0.00225	20.0	MF	tpsA,tpsB
GO:0070290	N-acylphosphatidylethanolamine-specific ...	7	3	0.33	0.00323	9.1	MF	DDB_G0271270,DDB_G0293392,pldB
GO:0005543	phospholipid binding	48	7	2.28	0.00706	3.1	MF	dagA,DDB_G0274273,DDB_G0280921,hipA,nxnA,tom1,vps5
GO:0035251	UDP-glucosyltransferase activity	10	3	0.48	0.00996	6.3	MF	tpsA,tpsB,ugt52
GO:0006970	response to osmotic stress	109	22	5.09	4.10E-09	4.3	BP	abcB1,abcG21,abpC,act1,act19,cprA,cupA,cupB,cupC,cupF,cupG, cupH,cupI,dstC,dymB,mhcA,p2xC,rtoA,sigJ, 3 DDB_G genes
GO:0009651	response to salt stress	13	8	0.61	2.20E-08	13.1	BP	cupA,cupB,cupC,cupF,cupG,cupH,cupI,dstC
GO:0055114	oxidation-reduction process	554	52	25.89	7.10E-07	2.0	BP	aco2,aifB,alrE,chdh,cshA,cxgS,cyp508A4,cyp515B1,cyp516B1,iliB, malA,maoB-1,msrA,noxB,rnrA, 37 DDB_G genes
GO:0009262	deoxyribonucleotide metabolic process	8	4	0.37	0.00028	10.8	BP	dak,DDB_G0293580,dut,rnrA
GO:0010564	regulation of cell cycle process	24	6	1.12	0.00066	5.4	BP	aurK,bub1,cdc45,DDB_G0273201,DDB_G0291842,ube2c
GO:0009408	response to heat	11	4	0.51	0.00119	7.8	BP	DDB_G0291314,dnaja1,dstC,rtoA
GO:0008202	steroid metabolic process	28	6	1.31	0.00157	4.6	BP	cas1,DDB_G0270946,DDB_G0277203,DDB_G0282197,erg2,ugt52
GO:0045165	cell fate commitment	6	3	0.28	0.00182	10.7	BP	btg,detA,zak2
GO:1901987	regulation of cell cycle phase transitio...	13	4	0.61	0.0024	6.6	BP	bub1,cdc45,DDB_G0273201,ube2c
GO:0006271	DNA strand elongation involved in DNA re...	14	4	0.65	0.00323	6.2	BP	lig1,polA1,repG,rfc3
GO:0009405	pathogenesis	14	4	0.65	0.00323	6.2	BP	DDB_G0267792,prtA,prtB,uduD1
GO:0005992	trehalose biosynthetic process	3	2	0.14	0.00633	14.3	BP	tpsA,tpsB
GO:0044786	cell cycle DNA replication	3	2	0.14	0.00633	14.3	BP	cdc45,lig1
GO:0007091	metaphase/anaphase transition of mitotic...	10	3	0.47	0.0095	6.4	BP	bub1,DDB_G0273201,ube2c
GO:0006261	DNA-dependent DNA replication	40	6	1.87	0.00998	3.2	BP	cdc45,lig1,mcm4,polA1,repG,rfc3
GO:0032133	chromosome passenger complex	2	2	0.09	0.0022	22.2	CC	aurK,icpA
GO:0000922	spindle pole	10	3	0.47	0.0096	6.4	CC	aurK,icpA,plk

B)

GO.ID	Term	Annotated	Significant	Expected	classic	fold enrichment	categ	genes
GO:0004531	deoxyribonuclease II activity	11	3	0.16	0.00044	18.8	MF	DDB_G0270850,DDB_G0270958,dnase2
GO:0098599	palmitoyl hydrolase activity	3	2	0.04	0.00061	50.0	MF	ppt2,ppt3
GO:0016798	hydrolase activity, acting on glycosyl b...	103	7	1.49	0.00067	4.7	MF	alyD-1,athl1,DDB_G0274181,DDB_G0287465,DDB_G0293566,sgmA, sgmB
GO:0003796	lysozyme activity	13	3	0.19	0.00075	15.8	MF	alyD-1,DDB_G0274181,DDB_G0293566
GO:0004180	carboxypeptidase activity	5	2	0.07	0.002	28.6	MF	DDB_G0280105,DDB_G0291912
GO:0004767	sphingomyelin phosphodiesterase activity	5	2	0.07	0.002	28.6	MF	sgmA,sgmB
GO:0046943	carboxylic acid transmembrane transporte...	21	3	0.3	0.00321	10.0	MF	DDB_G0284051,DDB_G0287303,mcfZ
GO:0004620	phospholipase activity	22	3	0.32	0.00367	9.4	MF	plbE,sgmA,sgmB
GO:0017171	serine hydrolase activity	49	4	0.71	0.00525	5.6	MF	DDB_G0280105,DDB_G0283989,DDB_G0290409,DDB_G0291912
GO:0006012	galactose metabolic process	6	4	0.09	7.50E-07	44.4	BP	DDB_G0282525,galE,galK,uppA
GO:0015939	pantothenate metabolic process	2	2	0.03	0.00023	66.7	BP	DDB_G0286637,panC
GO:0006022	aminoglycan metabolic process	23	4	0.35	0.00036	11.4	BP	alyD-1,DDB_G0274181,DDB_G0287465,DDB_G0293566
GO:0044242	cellular lipid catabolic process	25	4	0.38	0.0005	10.5	BP	DDB_G0277477,plbE,sgmA,sgmB
GO:0006309	apoptotic DNA fragmentation	11	3	0.17	0.00052	17.6	BP	DDB_G0270850,DDB_G0270958,dnase2
GO:0009395	phospholipid catabolic process	11	3	0.17	0.00052	17.6	BP	plbE,sgmA,sgmB
GO:0098734	macromolecule depalmitoylation	3	2	0.05	0.00068	40.0	BP	ppt2,ppt3
GO:0006564	L-serine biosynthetic process	4	2	0.06	0.00135	33.3	BP	serA,serC
GO:0006685	sphingomyelin catabolic process	4	2	0.06	0.00135	33.3	BP	sgmA,sgmB
GO:0042742	defense response to bacterium	23	3	0.35	0.00487	8.6	BP	alyD-1,DDB_G0274181,DDB_G0293566
GO:0015711	organic anion transport	27	3	0.41	0.0077	7.3	BP	DDB_G0287303,DDB_G0292424,mcfZ
GO:1901071	glucosamine-containing compound metaboli...	10	2	0.15	0.00955	13.3	BP	DDB_G0287465,nagB1
GO:0005778	peroxisomal membrane	22	3	0.33	0.00407	9.1	CC	ddo-2,mcfQ,pex13
GO:0005777	peroxisome	50	4	0.75	0.0064	5.3	CC	DDB_G0277477,ddo-2,mcfQ,pex13

S3 Table: Selected gene ontology (GO) enrichment data of differentially expressed genes in response to short exposure to curcumin.

A) 533 genes were identified as up-regulated upon early exposure (4 hours) to high concentration (10 μg/ml) of curcumin. GO enrichment analysis revealed the genes are involved in various functions including oxidoreductase activity, response to osmotic/salt/heat stress and the cell cycle. Eleven ABC transporters are also included. B) 145 genes were identified as down-regulated upon early exposure (4 hours) to high concentration (10 μg/ml) of curcumin. GO analysis revealed the genes are involved in functions including hydrolase activity, sphingomyelin catabolism, apoptosis, defense response to bacterium, and peroxisome function.
