# Supplementary material for: Curcumin affects gene expression and reactive oxygen species via a PKA dependent mechanism in Dictyostelium discoideum
Source: PLoS One. 2017 Nov 14;12(11):e0187562. doi: 10.1371/journal.pone.0187562 (PMC5685611; doi:10.1371/journal.pone.0187562)
Supplement: S4 Table — A) 204 genes up-regulated upon extended exposure (12 hours) to high concentration (10 μg/ml) of curcumin are involved in various functions including oxidoreductase activity, antioxidant activity, vitamin binding, response to abiotic stimulus, and contractile vacuole. Seven ABC transporters and 6 transcription factors including STATb and STATc, are also included. B) 443 genes down-regulated upon extended exposure to curcumin are involved in various functions including cell cycle control, DNA replication and responses to drugs. Eight genes that encode cytochrome P450 family proteins, which generally have a terminal oxidoreductase activity, are also included. Note that carA-1, pkaR and regA, which are involved in cAMP-mediated signaling, are also down-regulated upon extended exposure. (PDF) [file pone.0187562.s005.pdf]

A)

GO.ID	Term	Annotated	Significant	Expected	classic	fold enrichment	categ	genes
GO:0016491	oxidoreductase activity	513	33	10.18	8.00E-10	3.2	MF	aifB,ccs,cyp508A4,cyp516B1,hpdl-1,ndrJ,scdA,sqrdl,25 DDB_G genes
GO:0042626	ATPase activity, coupled to transmembran...	92	8	1.83	0.00045	4.4	MF	abcB1,abcG11,abcG12,abcG15,abcG17-1,abcG21,abcG9,patA
GO:0003700	transcription factor activity, sequence-...	66	6	1.31	0.00187	4.6	MF	bzpP,dimB,dstB,dstC,gtaE,stkA
GO:0031419	cobalamin binding	4	2	0.08	0.00229	25.0	MF	mut,ndrJ
GO:0016209	antioxidant activity	32	4	0.64	0.00347	6.3	MF	ccs,DDB_G0268192,DDB_G0269270,DDB_G0272280
GO:0022857	transmembrane transporter activity	263	12	5.22	0.00586	2.3	MF	abcB1,abcG11,abcG12,abcG15,abcG17-1,abcG21,abcG9,patA,rhgB ,symA,DDB_G0271672,DDB_G0283629
GO:0019842	vitamin binding	20	3	0.4	0.00681	7.5	MF	DDB_G0287671,mut,ndrJ
GO:0055114	oxidation-reduction process	554	33	10.73	3.40E-09	3.1	BP	aifB,ccs,cyp508A4,cyp516B1,hpdl-1,ndrJ,scdA,sqrdl,25 DDB_G genes
GO:0009628	response to abiotic stimulus	143	8	2.77	0.0064	2.9	BP	abcB1,abcG21,dnaja1,dstC,dymB,mgp1,phgA,sigJ
GO:0042127	regulation of cell proliferation	8	2	0.15	0.0097	13.3	BP	DDB_G0267706,dstB
GO:0000331	contractile vacuole	47	5	0.9	0.0019	5.6	CC	dymB,hipA,mgp1,patA,phgA

B)

GO.ID	Term	Annotated	Significant	Expected	classic	fold enrichment	categ	genes
GO:0016798	hydrolase activity, acting on glycosyl b...	103	15	4.4	2.70E-05	3.4	MF	athl1,chid1,ctbsA,ctbsB,iliG,parG,rliF, 8 DDB_G
GO:0003896	DNA primase activity	3	3	0.13	7.70E-05	23.1	MF	polA1,polA3,polA4
GO:0019239	deaminase activity	12	5	0.51	8.50E-05	9.8	MF	ada,cda,DDB_G0275179,ftcd,guaD
GO:0008061	chitin binding	8	4	0.34	0.0002	11.8	MF	chid1,ctbsA,ctbsB,DDB_G0268090
GO:0016712	oxidoreductase activity, acting on paire...	20	5	0.85	0.00125	5.9	MF	cyp508A2-1,cyp508A3-1,cyp508B1,cyp518A1,cyp519E1
GO:0005044	scavenger receptor activity	2	2	0.09	0.00182	22.2	MF	DDB_G0284479,dduF
GO:0099600	transmembrane receptor activity	54	8	2.31	0.00188	3.5	MF	carA-1,fslB,fslH,grlB,grlG,grlJ,p2xA,p2xE
GO:0020037	heme binding	72	9	3.07	0.00333	2.9	MF	cyp508A2-1,cyp508A3-1,cyp508B1,cyp513A1,cyp513A3,cyp513C1, cyp518A1,cyp519E1,DDB_G0294553
GO:0016841	ammonia-lyase activity	8	3	0.34	0.00368	8.8	MF	ftcd,hal,sds
GO:1990939	ATP-dependent microtubule motor activity	8	3	0.34	0.00368	8.8	MF	kif13,kif2,kif5
GO:0003688	DNA replication origin binding	3	2	0.13	0.0053	15.4	MF	cdc45,mcm5
GO:0016840	carbon-nitrogen lyase activity	11	3	0.47	0.00984	6.4	MF	ftcd,hal,sds
GO:0006260	DNA replication	75	17	3.42	2.80E-08	5.0	BP	cdc45,gins4,mcm5,orcC,pds5,polA1,polA2,polA3,polA4,polD2,polD3, repG,rfc4,rnrA,rnrB-1,smc3,top2
GO:0007067	mitotic nuclear division	69	12	3.14	5.50E-05	3.8	BP	bub1,bub3,cdc20,cdk1,DDB_G0273201,icpA,kif13,kif2,ncapGa,pds5, smc1,smc3
GO:0006269	DNA replication, synthesis of RNA primer	3	3	0.14	9.40E-05	21.4	BP	polA1,polA3,polA4
GO:0042493	response to drug	27	7	1.23	0.00015	5.7	BP	crtp3,cyp508A2-1,cyp508A3-1,cyp508B1,cyp518A1,cyp519E1,fps
GO:0000819	sister chromatid segregation	29	7	1.32	0.00025	5.3	BP	bub1,DDB_G0273201,kif2,ncapGa,smc1,smc3,top2
GO:0006030	chitin metabolic process	8	4	0.36	0.00026	11.1	BP	chid1,ctbsA,ctbsB,DDB_G0268090
GO:0023058	adaptation of signaling pathway	4	3	0.18	0.00036	16.7	BP	adcB,adcC,carA-1
GO:1901072	glucosamine-containing compound cataboli...	9	4	0.41	0.00044	9.8	BP	chid1,ctbsA,ctbsB,DDB_G0268090
GO:0006270	DNA replication initiation	11	4	0.5	0.00108	8.0	BP	cdc45,gins4,mcm5,polA1
GO:0042737	drug catabolic process	20	5	0.91	0.00167	5.5	BP	cyp508A2-1,cyp508A3-1,cyp508B1,cyp518A1,cyp519E1
GO:0009164	nucleoside catabolic process	6	3	0.27	0.00169	11.1	BP	ada,cda,DDB_G0275179
GO:0002031	G-protein coupled receptor internalizati...	2	2	0.09	0.00207	22.2	BP	adcB,adcC
GO:0043949	regulation of cAMP-mediated signaling	8	3	0.36	0.00442	8.3	BP	adcB,adcC,pkaR
GO:0000727	double-strand break repair via break-ind...	3	2	0.14	0.00602	14.3	BP	cdc45,gins4
GO:0007093	mitotic cell cycle checkpoint	9	3	0.41	0.00641	7.3	BP	bub1,DDB_G0273201,top2
GO:0046434	organophosphate catabolic process	27	5	1.23	0.00671	4.1	BP	Dd5P4,dut,pde3,plbB,regA
GO:0043596	nuclear replication fork	10	5	0.43	3.00E-05	11.6	CC	cdc45,gins4,polA1,polA3,polA4
GO:0000793	condensed chromosome	29	6	1.25	0.00123	4.8	CC	aurK,DDB_G0273201,icpA,ncapGa,smc1,smc3
GO:0030894	replisome	6	3	0.26	0.00143	11.5	CC	polA1,polA3,polA4
GO:0008278	cohesin complex	2	2	0.09	0.00184	22.2	CC	smc1,smc3
GO:0051233	spindle midzone	2	2	0.09	0.00184	22.2	CC	aurK,icpA
GO:0044454	nuclear chromosome part	47	7	2.02	0.00361	3.5	CC	aurK,cdc45,gins4,orcC,polA1,polA3,polA4
GO:0005875	microtubule associated complex	29	5	1.25	0.00719	4.0	CC	aurK,icpA,kif13,kif2,kif5

S4 Table: Selected GO enrichment data of differentially expressed genes upon extended exposure to curcumin.

A) 204 genes up-regulated upon extended exposure (12 hours) to high concentration (10 μg/ml) of curcumin are involved in various functions including oxidoreductase activity, antioxidant activity, vitamin binding, response to abiotic stimulus, and contractile vacuole. Seven ABC transporters and 6 transcription factors including STATb and STATc, are also included. B) 443 genes down-regulated upon extended exposure to curcumin are involved in various functions including cell cycle control, DNA replication and responses to drugs. Eight genes that encode cytochrome P450 family proteins, which generally have a terminal oxidoreductase activity, are also included. Note that *carA-1*, *pkaR* and *regA*, which are involved in cAMP-mediated signaling, are also down-regulated upon extended exposure.
