# Supplementary material for: Curcumin affects gene expression and reactive oxygen species via a PKA dependent mechanism in Dictyostelium discoideum
Source: PLoS One. 2017 Nov 14;12(11):e0187562. doi: 10.1371/journal.pone.0187562 (PMC5685611; doi:10.1371/journal.pone.0187562)
Supplement: S5 Table — A) 67 genes are up-regulated during growth without exposure to curcumin. The GO term enrichment analysis revealed mostly cell cycle related genes. B) 23 genes are down-regulated during growth without exposure to curcumin. (PDF) [file pone.0187562.s006.pdf]

A)

GO.ID	Term	Annotated	Significant	Expected	classic	fold enrichment	categ	genes
GO:0003796	lysozyme activity	13	2	0.09	0.0031	22.2	MF	alyB,alyC
GO:0016491	oxidoreductase activity	513	9	3.37	0.0054	2.7	MF	aifC,alrE,chdh,cxgS,cyp517A2,DDB_G0277203,DDB_G0283653, DDB_G0294589,DDB_G0295807
GO:0003887	DNA-directed DNA polymerase activity	19	2	0.12	0.0067	16.7	MF	polA1,polA2
GO:0016818	hydrolase activity, acting on acid anhyd...	447	8	2.94	0.0079	2.7	MF	abcA7,abcA8,cdc45,DDB_G0269664,ddx42,kif13,rabR,wrnip1
GO:0050830	defense response to Gram-positive bacter...	5	2	0.03	0.00043	66.7	BP	alyB,alyC
GO:0070534	protein K63-linked ubiquitination	5	2	0.03	0.00043	66.7	BP	DDB_G0288381,DDB_G0288697
GO:0010564	regulation of cell cycle process	24	3	0.16	0.00051	18.8	BP	aurK,bub1,cdc45
GO:0007067	mitotic nuclear division	69	4	0.46	0.00111	8.7	BP	bub1,cdc20,kif13,smc2
GO:0098813	nuclear chromosome segregation	34	3	0.23	0.00145	13.0	BP	bub1,kif13,smc2
GO:0006260	DNA replication	75	4	0.5	0.00151	8.0	BP	cdc45,polA1,polA2,wrnip1
GO:0006270	DNA replication initiation	11	2	0.07	0.00231	28.6	BP	cdc45,polA1
GO:0006301	postreplication repair	11	2	0.07	0.00231	28.6	BP	DDB_G0288381,DDB_G0288697
GO:1901987	regulation of cell cycle phase transitio...	13	2	0.09	0.00325	22.2	BP	bub1,cdc45
GO:0051301	cell division	156	5	1.04	0.00366	4.8	BP	aurK,cdc20,DDB_G0280249,kif13,smc2
GO:0000270	peptidoglycan metabolic process	14	2	0.09	0.00378	22.2	BP	alyB,alyC
GO:0006281	DNA repair	159	5	1.06	0.00397	4.7	BP	cdc45,DDB_G0288381,DDB_G0288697,polA1,wrnip1
GO:0000209	protein polyubiquitination	19	2	0.13	0.00695	15.4	BP	DDB_G0288381,DDB_G0288697
GO:0031983	vesicle lumen	5	2	0.03	0.00043	66.7	CC	alyB,alyC
GO:0005694	chromosome	119	5	0.8	0.00109	6.3	CC	aurK,cdc45,DDB_G0293620,polA1,smc2
GO:0043596	nuclear replication fork	10	2	0.07	0.0019	28.6	CC	cdc45,polA1
GO:0000228	nuclear chromosome	54	3	0.36	0.00544	8.3	CC	aurK,cdc45,polA1
GO:0000775	chromosome, centromeric region	22	2	0.15	0.00926	13.3	CC	aurK,DDB_G0293620

B)

GO.ID	Term	Annotated	Significant	Expected	classic	fold enrichment	categ	genes
GO:0022414	reproductive process	102	3	0.25	0.0017	12.0	BP	DDB_G0270366,DDB_G0270368,msh4
GO:0044703	multi-organism reproductive process	37	2	0.09	0.0035	22.2	BP	DDB_G0270366,DDB_G0270368

S5 Table: Selected GO enrichment data of differentially expressed genes during growth without exposure to curcumin.

A) 67 genes are up-regulated during growth without exposure to curcumin. The GO term enrichment analysis revealed mostly cell cycle related genes. B) 23 genes are down-regulated during growth without exposure to curcumin.
