# Supplementary material for: Curcumin affects gene expression and reactive oxygen species via a PKA dependent mechanism in Dictyostelium discoideum
Source: PLoS One. 2017 Nov 14;12(11):e0187562. doi: 10.1371/journal.pone.0187562 (PMC5685611; doi:10.1371/journal.pone.0187562)
Supplement: S6 Table — A) Genes up-regulated without curcumin (S5 Table) were omitted from the GO term enrichment analysis to focus on genes up-regulated due to curcumin exposure during growth. 161 genes were up-regulated at 12 hours only under curcumin treatment. Genes related to oxidoreductase activity, gultathione transferase activity, oxygen transport, response to oxidative stress, and sphingolipid catabolic process were enriched in the gene set. B) 733 genes were down-regulated under curcumin treatment. Genes related to DNA replication, oxidoreductase activity and contractile vacuolar membrane, are enriched in the gene set. Genes that encode cytochrome P450 family proteins, which generally have a terminal oxidoreductase activity, and genes involved in cAMP-mediated signaling, carA-1, pkaR and regA, are also down-regulated upon extended exposure. (PDF) [file pone.0187562.s007.pdf]

A)

GO.ID	Term	Annotated	Significant	Expected	classic	fold enrichment	categ	genes
GO:0016298	lipase activity	35	6	0.58	2.10E-05	10.3	MF	DDB_G0277473,DDB_G0291354,plbA,plbE,sgmA,sgmB
GO:0016708	oxidoreductase activity, acting on paire...	2	2	0.03	0.00028	66.7	MF	fhbA,fhbB
GO:0004620	phospholipase activity	22	4	0.37	0.00043	10.8	MF	plbA,plbE,sgmA,sgmB
GO:0004364	glutathione transferase activity	10	3	0.17	0.0005	17.6	MF	DDB_G0272632,DDB_G0280317,mai
GO:0042409	caffeoyl-CoA O-methyltransferase activit...	3	2	0.05	0.00082	40.0	MF	omt5,omt6
GO:0046915	transition metal ion transmembrane trans...	14	3	0.23	0.00145	13.0	MF	mcfF,nramp1,p80
GO:0004185	serine-type carboxypeptidase activity	4	2	0.07	0.00162	28.6	MF	DDB_G0280105,DDB_G0291912
GO:0004767	sphingomyelin phosphodiesterase activity	5	2	0.08	0.00268	25.0	MF	sgmA,sgmB
GO:0005381	iron ion transmembrane transporter activ...	7	2	0.12	0.0055	16.7	MF	mcfF,nramp1
GO:0016798	hydrolase activity, acting on glycosyl b...	103	6	1.72	0.00733	3.5	MF	alyD-1,DDB_G0270074,DDB_G0287465,DDB_G0293566,sgmA,sgmB
GO:0030170	pyridoxal phosphate binding	48	4	0.8	0.00817	5.0	MF	agxt,cysB,hemA,serC
GO:0051188	cofactor biosynthetic process	89	12	1.6	5.20E-08	7.5	BP	accA,coq7,coxA,DDB_G0285011,DDB_G0286637,DDB_G0290401, fol1,gchA,hemA,hemF,panC,ptsA
GO:0009110	vitamin biosynthetic process	8	4	0.14	6.70E-06	28.6	BP	DDB_G0286637,fol1,panC,ptsA
GO:0009395	phospholipid catabolic process	11	4	0.2	3.00E-05	20.0	BP	plbA,plbE,sgmA,sgmB
GO:0009396	folic acid-containing compound biosynthe...	8	3	0.14	0.0003	21.4	BP	fol1,gchA,ptsA
GO:0015940	pantothenate biosynthetic process	2	2	0.04	0.00032	50.0	BP	DDB_G0286637,panC
GO:0009070	serine family amino acid biosynthetic pr...	9	3	0.16	0.00044	18.8	BP	cysB,serA,serC
GO:0006575	cellular modified amino acid metabolic p...	21	4	0.38	0.00048	10.5	BP	fol1,gchA,pssA,ptsA
GO:0006732	coenzyme metabolic process	104	8	1.87	0.00055	4.3	BP	accA,coq7,DDB_G0286637,DDB_G0290401,fol1,gchA,panC,ptsA
GO:0006783	heme biosynthetic process	10	3	0.18	0.00063	16.7	BP	coxA,hemA,hemF
GO:0006563	L-serine metabolic process	11	3	0.2	0.00085	15.0	BP	cysB,serA,serC
GO:0015671	oxygen transport	3	2	0.05	0.00096	40.0	BP	fhbA,fhbB
GO:0046655	folic acid metabolic process	3	2	0.05	0.00096	40.0	BP	fol1,ptsA
GO:0033014	tetrapyrrole biosynthetic process	12	3	0.22	0.00112	13.6	BP	coxA,hemA,hemF
GO:0006979	response to oxidative stress	69	6	1.24	0.00145	4.8	BP	alrA,aoxA,cysB,DDB_G0275311,gchA,ptsA
GO:0006684	sphingomyelin metabolic process	4	2	0.07	0.00189	28.6	BP	sgmA,sgmB
GO:0006826	iron ion transport	4	2	0.07	0.00189	28.6	BP	mcfF,nramp1
GO:0030149	sphingolipid catabolic process	6	2	0.11	0.00462	18.2	BP	sgmA,sgmB
GO:0044655	phagosome reneutralization	6	2	0.11	0.00462	18.2	BP	ccdc53,myoB
GO:0009607	response to biotic stimulus	87	6	1.57	0.0047	3.8	BP	alrA,alyD-1,DDB_G0293566,myoB,nramp1,omt6
GO:0006026	aminoglycan catabolic process	22	3	0.4	0.00687	7.5	BP	alyD-1,DDB_G0287465,DDB_G0293566
GO:0045335	phagocytic vesicle	341	20	6.13	2.20E-06	3.3	CC	acly,alrA,ccdc53,ddcB,fhbA,fhbB,gchA,gp130,myoB,nagB1,nramp1, omt6,sdrA,serA, 6 DDB_G genes

B)

GO.ID	Term	Annotated	Significant	Expected	classic	fold enrichment	categ	genes
GO:0003689	DNA clamp loader activity	6	5	0.41	8.10E-06	12.2	MF	dcc1,rfc2,rfc3,rfc4,rfc5
GO:0003896	DNA primase activity	3	3	0.2	0.00031	15.0	MF	polA1,polA3,polA4
GO:0016502	nucleotide receptor activity	13	5	0.88	0.00116	5.7	MF	carA-1,crlC,p2xA,p2xB,p2xE
GO:0016863	intramolecular oxidoreductase activity, ...	4	3	0.27	0.00118	11.1	MF	ebp,ech1,erg2
GO:0005044	scavenger receptor activity	2	2	0.14	0.00461	14.3	MF	DDB_G0284479,dduF
GO:0015278	calcium-release channel activity	6	3	0.41	0.00534	7.3	MF	p2xA,p2xB,p2xE
GO:0015085	calcium ion transmembrane transporter ac...	12	4	0.82	0.00672	4.9	MF	DDB_G0289473,p2xA,p2xB,p2xE
GO:0016712	oxidoreductase activity, acting on paire...	20	5	1.36	0.00937	3.7	MF	cyp508A4,cyp515B1,cyp516B1,cyp518A1,cyp519E1
GO:0006260	DNA replication	75	28	5.27	3.50E-14	5.3	BP	cdc45,dcc1,DDB_G0283189,DDB_G0293864,gins1,gins4,lig1,mcm4, mcm5,mcm7,pds5,polA1,polA2,polA3,polA4,polD2,polD3,repG,rfc2,rfc3, rfc4,rfc5,rnrA,rnrB-1,smc3,ssrp1,top2,wrnip1
GO:0006261	DNA-dependent DNA replication	40	16	2.81	3.60E-09	5.7	BP	cdc45,DDB_G0283189,gins4,lig1,mcm4,mcm5,mcm7,polA1,polA3, polA4,repG,rfc2,rfc3,rfc4,rfc5,top2
GO:0000819	sister chromatid segregation	29	12	2.04	2.20E-07	5.9	BP	anapc10,bub1,dcc1,DDB_G0273201,kif2,ncapD3,ncapGa,smc1,smc2, smc3,top2,ube2c
GO:0006271	DNA strand elongation involved in DNA re...	14	8	0.98	1.20E-06	8.2	BP	DDB_G0283189,lig1,polA1,repG,rfc2,rfc3,rfc4,rfc5
GO:0006970	response to osmotic stress	109	22	7.66	5.10E-06	2.9	BP	abcG21,act1,act20,act6,cpnA,cprA,cupB,cupC,cupF,cupG,cupI,dstC, p2xA,p2xB,p2xE,regA,rtoA,sigJ,vwkA, 3 DDB_G genes
GO:0006270	DNA replication initiation	11	6	0.77	4.00E-05	7.8	BP	cdc45,gins4,mcm4,mcm5,mcm7,polA1
GO:0009651	response to salt stress	13	6	0.91	0.00013	6.6	BP	cupB,cupC,cupF,cupG,cupI,dstC
GO:0009405	pathogenesis	14	6	0.98	0.00022	6.1	BP	DDB_G0267792,prtA,prtB,uduD1,uduD2,uduF
GO:0006816	calcium ion transport	15	6	1.05	0.00034	5.7	BP	DDB_G0284777,DDB_G0289473,p2xA,p2xB,p2xE,regA
GO:0006269	DNA replication, synthesis of RNA primer	3	3	0.21	0.00034	14.3	BP	polA1,polA3,polA4
GO:0007091	metaphase/anaphase transition of mitotic...	10	4	0.7	0.00359	5.7	BP	anapc10,bub1,DDB_G0273201,ube2c
GO:0009408	response to heat	11	4	0.77	0.00533	5.2	BP	DDB_G0282313,dnaja1,dstC,rtoA
GO:0007076	mitotic chromosome condensation	7	3	0.49	0.00974	6.1	BP	ncapD3,ncapGa,smc2
GO:0050848	regulation of calcium-mediated signaling	7	3	0.49	0.00974	6.1	BP	p2xA,p2xB,p2xE
GO:0005657	replication fork	16	10	1.14	1.60E-08	8.8	CC	cdc45,DDB_G0283189,gins4,polA1,polA3,polA4,rfc2,rfc3,rfc4,rfc5
GO:0000775	chromosome, centromeric region	22	9	1.57	9.30E-06	5.7	CC	aurK,bub3,dcc1,DDB_G0273201,DDB_G0293620,hcpA,hcpB,icpA,kif4
GO:0009986	cell surface	39	11	2.78	5.70E-05	4.0	CC	aurK,carA-1,csaA,csbA,csbB,dlpA,dscA-1,dscD-1,gmsA,icpA,sigB
GO:0030894	replisome	6	4	0.43	0.00034	9.3	CC	DDB_G0283189,polA1,polA3,polA4
GO:0005658	alpha DNA polymerase:primase complex	3	3	0.21	0.00036	14.3	CC	polA1,polA3,polA4
GO:0031261	DNA replication preinitiation complex	5	3	0.36	0.00321	8.3	CC	cdc45,gins1,gins4
GO:0000793	condensed chromosome	29	7	2.06	0.00348	3.4	CC	aurK,DDB_G0273201,icpA,ncapGa,smc1,smc2,smc3
GO:0005720	nuclear heterochromatin	2	2	0.14	0.00505	14.3	CC	hcpA,hcpB
GO:0008278	cohesin complex	2	2	0.14	0.00505	14.3	CC	smc1,smc3
GO:0033118	esterosome membrane	2	2	0.14	0.00505	14.3	CC	cryS,D2
GO:0051233	spindle midzone	2	2	0.14	0.00505	14.3	CC	aurK,icpA
GO:1990077	primosome complex	2	2	0.14	0.00505	14.3	CC	polA3,polA4
GO:0031164	contractile vacuolar membrane	19	5	1.35	0.009	3.7	CC	cpnA,p2xA,p2xB,p2xE,vwkA

S6 Table: Selected GO enrichment data of genes which are differentially expresssed during growth only under curcumin treatment.

A) Genes up-regulated without curcumin (S5 Table) were omitted from the GO term enrichment analysis to focus on genes up-regulated due to curcumin exposure during growth. 161 genes were up-regulated at 12 hours only under curcumin treatment. Genes related to oxidoreductase activity, gultathione transferase activity, oxygen transport, response to oxidative stress, and sphingolipid catabolic process were enriched in the gene set. B) 733 genes were down-regulated under curcumin treatment. Genes related to DNA replication, oxidoreductase activity and contractile vacuolar membrane, are enriched in the gene set. Genes that encode cytochrome P450 family proteins, which generally have a terminal oxidoreductase activity, and genes involved in cAMP-mediated signaling, *carA-1*, *pkaR* and *regA*, are also down-regulated upon extended exposure.
