# Supplementary material for: Curcumin affects gene expression and reactive oxygen species via a PKA dependent mechanism in Dictyostelium discoideum
Source: PLoS One. 2017 Nov 14;12(11):e0187562. doi: 10.1371/journal.pone.0187562 (PMC5685611; doi:10.1371/journal.pone.0187562)
Supplement: S7 Table — Among the genes that are differentially expressed only under curcumin treatment (S6 Table), 50 genes were always expressed at higher levels (at least 2 fold) in the cells treated with high concentration (10 μg/ml) of curcumin. Genes involved in antioxidant activity were highly enriched. Note that most of the genes exhibited peak expression at an early time point (4 hours, refer to Fig 12E). (PDF) [file pone.0187562.s008.pdf]

GO.ID	Term	Annotated	Significant	Expected	classic	fold enrichment	categ	genes
GO:0016491	oxidoreductase activity	513	12	2.27	8.20E-07	5.3	MF	cyp516B1,11 DDB_G genes
GO:0016209	antioxidant activity	32	3	0.14	0.00036	21.4	MF	DDB_G0268192,DDB_G0269270,DDB_G0272280
GO:0055114	oxidation-reduction process	554	12	2.59	4.10E-06	4.6	BP	cyp516B1,11 DDB_G genes
GO:0009408	response to heat	11	2	0.05	0.0011	40.0	BP	dnaja1,dstC
GO:0006972	hyperosmotic response	57	3	0.27	0.0023	11.1	BP	abcG21,dstC,sigJ
GO:0009266	response to temperature stimulus	19	2	0.09	0.0035	22.2	BP	dnaja1,dstC
GO:0009628	response to abiotic stimulus	143	4	0.67	0.0042	6.0	BP	abcG21,dnaja1,dstC,sigJ

S7 Table: Selected GO enrichment data of up-regulated genes at all time points under curcumin treatment.

Among the genes that are differentially expressed only under curcumin treatment (S6 Table), 50 genes were always expressed at higher levels (at least 2 fold) in the cells treated with high concentration (10 μg/ml) of curcumin. Genes involved in antioxidant activity were highly enriched. Note that most of the genes exhibited peak expression at an early time point (4 hours, Refer to Fig. 12E).
